# Dysregulation of Immune Mediators and Synaptic Plasticity in Central Nervous System Disorders

**DOI:** 10.3390/cells15020201

**Published:** 2026-01-21

**Authors:** Paola Imbriani, Clara D’Ambra, Roberta De Mori, Marta Ionta, Alessandro Renna, Paola Bonsi

**Affiliations:** Istituto di Ricovero e Cura a Carattere Scientifico (IRCCS) Fondazione Santa Lucia, 00143 Rome, Italy; p.imbriani@hsantalucia.it (P.I.); r.demori@hsantalucia.it (R.D.M.); martaionta@gmail.com (M.I.);

**Keywords:** neuroinflammation, microglia, astrocytes, cytokines, neuroplasticity, Alzheimer’s disease (AD), Parkinson’s disease (PD), autism spectrum disorder (ASD), multimodal stimulation, environmental enrichment

## Abstract

Bidirectional communication between the central nervous system and the immune system is crucial for brain function, particularly in regulating neuroplasticity: on the one hand, glial cells modulate neuronal function, brain circuitry, axon myelination, dendritic spine architecture, and information processing, while on the other hand, neuronal activity can alter the immune response. Neuroinflammation and dysregulation of astroglia and microglia can be detrimental to brain development and function. In particular, maladaptive responses and chronic glial activation have been correlated to synaptic dysfunction in diverse brain conditions. In the present review, we will provide a general introduction to the main players of the neuroimmune response and their ability to modulate neuroplasticity, followed by a comprehensive overview of experimental evidence linking the dysregulation of immune mediators to the disruption of synaptic plasticity in neurodegenerative and neurodevelopmental disorders, with a specific focus on Alzheimer’s disease, Parkinson’s disease, and autism spectrum disorder.

## 1. Introduction

For many years, the brain was considered an immune-privileged organ, largely isolated from the peripheral immune system by the blood–brain barrier (BBB). It is now well established that the central nervous system (CNS) and the immune system engage in continuous, bidirectional communication under both physiological and pathological conditions [1]. In the healthy brain, this interaction contributes to learning and memory by regulating neuroplasticity, which relies on activity-dependent modifications of synaptic strength and connectivity [2,3]. Synaptic plasticity was first described in the hippocampus, where repeated and synchronous activation of both pre- and postsynaptic neurons produces a selective increase in the strength of the stimulated synaptic input [4]. This phenomenon is known as long-term potentiation (LTP) and is induced by high-frequency tetanic stimulation in experimental conditions. However, in order to prevent the saturation of synaptic transmission long-term depression (LTD), an opposite form of synaptic plasticity, occurs in the brain as well. LTD is induced in the cortex by low-frequency tetanic stimulation [5] and it serves to reduce the effectiveness of synaptic connections allowing the continuous storage of new memories.

Glial cells, in particular microglia and astrocytes, play an active role in modulating synaptic plasticity; moreover, immune-related molecules, traditionally associated with host defense, are now recognized as modulators of synaptic efficacy and circuit remodeling [6]. On the other hand, in the CNS, neurons actively participate in immune regulation by influencing their glial cells counterparts and infiltrated T cells through both contact-dependent and contact-independent mechanisms. The first ones occur when, once the BBB integrity is impaired, neurons come into direct cell–cell contacts with activated microglia and T cells via several adhesion and/or recognition molecules (e.g., NCAM, CD47). The second ones are represented by soluble neuronal factors, including cytokines, neuropeptides, neurotrophins and neurotransmitters. In addition, neurons can promote apoptosis of activated microglia and T cells through their expression of Fas ligand. Through all these mechanisms, neurons try to counteract microglial and/or T cell activation and minimize the inflammatory damage [7].

When the finely tuned balance of the brain-immune interaction is disrupted, detrimental effects on synaptic homeostasis and neuronal function may occur. When brain-immune communication is overly amplified, it can lead to sustained neuroinflammation, directly damaging neurons and disrupting the critical balance necessary for normal brain function. Importantly, immune dysregulation does not necessarily initiate neurological disease, but frequently acts as a permissive or amplifying factor that accelerates synaptic dysfunction and disease progression.

Given these premises, in this review we will focus on how dysregulated immune mediators influence synaptic plasticity in some neurodegenerative and neurodevelopmental disorders. Rather than providing an exhaustive immunological overview, we adopted a neurobiological perspective centered on synaptic mechanisms, highlighting established pathways, emerging hypotheses, and unresolved questions. An extensive review of alterations in neuroimmune cross-talk encompassing all neurodegenerative and neurodevelopmental pathologies is beyond our scope, and we address the reader to other recent reviews for a more complete scenario [8,9,10,11,12].

## 2. Neuroimmune System and Neuroinflammation: The Main Players

The term “neuroinflammation” refers to the inflammatory response originated in the CNS after injury by infective or non-infective factors: it represents a specialized and tightly regulated immune response that differs substantially from peripheral immunity.

Systemic inflammatory triggers, such as exposure to lipopolysaccharides (LPS), viral infections, or other invading pathogens, can induce an acute neuroinflammatory response characterized by early activation of resident glial cells (microglia and astrocytes) and, under pathological conditions, infiltration of peripheral immune cells into the CNS [13]. However neuroinflammation can also be the consequence of stimuli other than infections, such as trauma, ischemia, toxic proteins, and neurodegenerative diseases [14].

Microglia and astrocytes play a central role in this process. By detecting stimuli through specific receptors, such as toll-like receptors (TLRs), receptor for advanced glycation end products (RAGE) and cyclic GMP–AMP synthase (cGAS), these cells secrete pro-inflammatory molecules, including cytokines, chemokines, lipid mediators, and nitric oxide (NO), to help recruit additional immune cells and promote damage containment. Pro-inflammatory mediators can also alter the BBB integrity by affecting endothelial tight junctions, thereby facilitating the conditional migration of T cells and macrophages from the periphery into the CNS [15]. The resulting acute neuroinflammatory response can be beneficial, as it is typically transient and self-limiting, contributing to damage containment and restoration of brain homeostasis with overall neuroprotective effects. In the healthy brain, after the stimulus is terminated, the resolution of inflammation depends on well-orchestrated innate and adaptive immune responses [16]. In contrast, prolonged persistence of the triggering stimulus, together with dysregulation of inflammatory pathways, impairs resolution mechanisms, with a switch from an acute to a chronic inflammatory state [17]. This results in strong and prolonged microglial activation, with overexpression of inflammatory molecules, tissue degeneration, and impaired integrity of the BBB with peripheral immune cell infiltration (lymphocytes and macrophages), further amplifying the release of inflammatory mediators.

Moreover, chronic neuroinflammation has been associated with impaired clearance of misfolded or damaged neuronal proteins, including tau and amyloid-β protein precursor (AβPP), potentially contributing to axonal transport deficits, paired helical filament formation, and synaptic dysfunction [18]. Sustained inflammation, arising from maladaptive neuroimmune interactions, can drive a self-perpetuating cycle of neuronal damage, contributing to the development of many brain diseases [18]. Whether these mechanisms act as primary drivers or as disease-contributing factors remains context- and disease stage-dependent.

**Figure 1:**
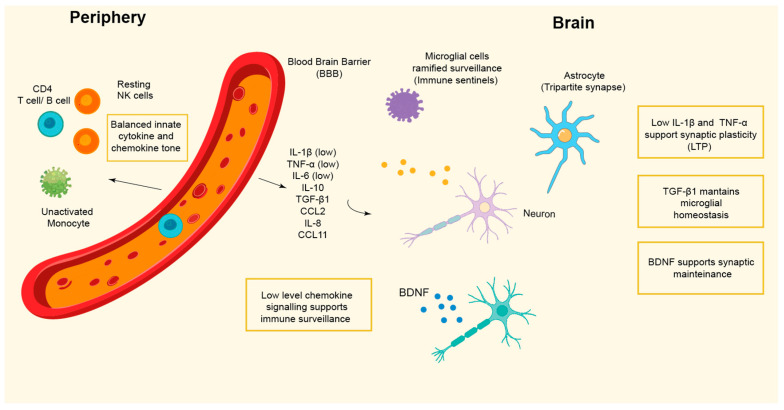
Homeostatic peripheral–central immune communication under physiological conditions. In the periphery, resting immune cells (CD4+ T cells, B cells, NK cells, and unactivated monocytes) contribute to a balanced innate cytokine and chemokine milieu. Low circulating levels of IL-1β, TNF-α, IL-6, IL-10, TGF-β1, CCL2, IL-8, and CCL11 cross or signal through the blood–brain barrier (BBB) to sustain central immune homeostasis. In the brain, microglia maintain a ramified “surveillance” state, supported by low-level chemokine signaling. Astrocytes and neurons integrate these cues to preserve synaptic function: low IL-1β and TNF-α promote long-term potentiation (LTP), TGF-β1 sustains microglial quiescence, and neuronal BDNF supports synaptic maintenance.

Multiple cells, including microglia, astrocytes, oligodendrocytes, and endothelial cells, are involved in sustaining the homeostasis of the CNS in non-pathological conditions, while dysregulation of their coordinated activity can exert detrimental effects on synaptic stability and neuronal integrity (Figure 1).

### 2.1. Microglia

Microglia are the resident immune cells of the CNS and represent its primary line of innate immune defense. These cells are derived from yolk-sac-derived macrophages during brain development and share several functional features with macrophages: they primarily use phagocytosis and cytotoxicity mechanisms to destroy degenerated cells and foreign materials. Microglia are widely distributed throughout the CNS, being found either adhered to neurons, near blood vessels, or inside the BBB [19]. Microglia are not truly “resting” even under physiological conditions, but continuously survey the brain, using their ramifications as “sentinels” of the surrounding microenvironment, and detect CNS damage. In the presence of an insult to the brain, they activate and orchestrate a specific immune response through processes including migration toward the site of the insult, phagocytosis, proliferation, presentation of antigens to T cells, and the release of a broad repertoire of bioactive mediators, including cytokines, chemokines, reactive oxygen and nitrogen species, complement proteins, and neurotrophic factors [20]. Microglial cells express TLRs for recognizing Damage-Associated Molecular Patterns (DAMPs), such as heat shock proteins and extracellular ATP, and Pathogen-Associated Molecular Patterns (PAMPs), such as bacterial LPS, thus initiating the innate immune response [8]. Activation of microglia can also promote the recruitment and engagement of adaptive immune responses under pathological conditions. Depending on their activation state, microglia can either trigger neuroinflammatory pathways leading to neurotoxicity and gradual neurodegeneration or promote downregulation of inflammation and neuroprotection.

Historically, microglial activation state has been described using a binary classification into a M1 pro-inflammatory phenotype and an M2 anti-inflammatory phenotype. The M1 state is characterized by the release of pro-inflammatory cytokines, such as interleukin-1 (IL-1), IL-6, tumor-necrosis factor-α (TNF-α), as well as reactive oxygen species (ROS) and NO, by which microglia recruit immune cells to escalate the immune response and initiate the activation of astrocytes; in contrast, the M2 phenotype is characterized by the secretion of growth factors and the release of anti-inflammatory cytokines, such as IL-10, IL-15, TGF-β, by which microglia can regulate their own deactivation after acute inflammation has been resolved [9].

This binary framework is now widely recognized as an oversimplification, as actually a continuum of different intermediate phenotypes between M1 and M2 can be recognized, and microglia can transit from one to another. In fact, single-cell RNA sequencing has revealed that microglia adopt highly heterogeneous and context-specific transcriptional states that vary with brain region, age, sex, and disease stage, such as disease-associated microglia (DAM), interferon-responsive microglia, neurodegeneration-associated microglia, and lipid-droplet-accumulating microglia [21]. This molecular and functional heterogeneity underscores the sophisticated regulatory role of microglia in the CNS, allowing them to fine-tune immune responses and modulate synaptic maintenance, remodeling, and plasticity in a context-dependent dynamic manner.

### 2.2. Astrocytes

Immune activation in the CNS also involves astrocytes, glial cells that, together with microglia, in non-pathological conditions contribute to sustain brain homeostasis and proper neuronal functioning. Astrocytes morphology varies depending on their development stage, subtype, and localization. Protoplasmic astrocytes in the gray matter exhibit short branches, while fibrous astrocytes within the white matter are distinguished by their elongated, unbranched cellular processes [22]. Analogous to microglial cells, astrocytes were traditionally classified into an A1, pro-inflammatory, neurotoxic phenotype, and an A2 neuroprotective phenotype [23]. However, similarly to the M1/M2 microglial framework, the A1/A2 dichotomy is now considered reductive, since transcriptomic studies have revealed a spectrum of reactive astrocyte states defined by diverse molecular signatures and disease-specific cues [24]. Notably, as primary mediators of CNS neuroinflammation, microglia can drive astrocytes into a reactive state by secreting pro-inflammatory cytokines, chemokines, TNF-α, and C1q. This activation modulates downstream target genes in astrocytes, prompting the release of factors that trigger a secondary neuroinflammatory cascade. On the other hand, astrocytic secretion of inflammatory mediators, such as monocyte chemoattractant protein-1 (MCP-1), CCL2 and CXCL10, can further amplify microglial activation and motility. This bidirectional communication extends beyond the glia, involving complex interactions with other CNS residents and infiltrating peripheral immune cells [25]. Thus, triggered by signals released by microglia during neuroinflammation, astrocytes can undergo morphological and functional changes characterized by hypertrophy, cytoskeletal remodeling (e.g., increased expression of glial fibrillary acid protein, GFAP), and the production of inflammatory cytokines, such as IL-1β and TNF-α, chemokines, prostaglandins, ROS, and complement factors, which influence surrounding neurons, microglia, and infiltrating immune cells [9]. Dysregulated astrocyte activation with ongoing release of pro-inflammatory mediators can contribute to chronic inflammation and neuronal damage. On the other hand, through the release of IL-4, IL-10, TGF-β and neurotrophic factors, astrocytes can also trigger neuroprotective response. Thus, reactive astrocytes can exert either neuroprotective or neurotoxic effects depending on temporal dynamics, environmental conditions, and the nature of the inflammatory stimulus. By functioning as a core element of the neurovascular unit, together with pericytes and endothelial cells, astrocytes are positioned to regulate vascular hemodynamics and the functional properties of the BBB [26]. Accordingly, astrocytes maintain ion and fluid balance through aquaporin-4 (AQP4)-mediated water transport, a process fundamental to the glymphatic clearance pathway. The concentration of these channels at the endfeet demonstrates a specialized astrocytic role in CNS waste removal [27].

Beyond their involvement in immune and inflammatory processes, astrocytes play a vital role in supporting synaptogenesis and synaptic transmission. Indeed, synaptic function and neuronal survival rely on astrocytic activity, as will be discussed later. Astrocytes dysfunction can compromise the metabolic support of neurons, limit the uptake of glutamate with increased neurotoxicity, and alter synaptic function, thus contributing to neuronal loss [28].

### 2.3. Peripheral Immune Cells

Peripheral immune cells contribute to CNS immune responses primarily under pathological conditions, when BBB integrity is compromised or permissive signaling pathways are activated. Neutrophils are among the first peripheral immune cells recruited to the CNS during acute inflammation. By releasing free radicals, proteolytic enzymes and matrix metalloproteinases, they contribute to impair the integrity of the BBB; moreover, activated neutrophils release Neutrophil Extracellular Traps (NETs), i.e., web-like structures composed of DNA, histones, and antimicrobial proteins derived from neutrophil degranulation, which further damage the BBB [29]. Beyond their antimicrobial role, neutrophils can indirectly influence synaptic environments by amplifying oxidative stress and promoting microglial activation, thereby contributing to synaptic instability during sustained inflammation [30].

Monocyte-derived macrophages are recruited from the periphery in response to CNS injury or neurodegeneration. Depending on the temporal phase of inflammation and local cues, these cells can exert either detrimental or reparative effects. Initially, infiltrating macrophages may release pro-inflammatory mediators that contribute to demyelination and tissue damage. At later stages, they can adopt phenotypes that support debris clearance, resolution of inflammation, and tissue repair through secretion of anti-inflammatory cytokines and neurotrophic factors [31]. These infiltrating macrophages interact closely with resident microglia, influencing their activation state and indirectly modulating synaptic remodeling during both injury and recovery phases [32].

Natural killer (NK) cells can access the CNS under inflammatory conditions in response to chemokine gradients. They produce pro-inflammatory cytokines, such as interferon-γ (IFN-γ), and cytotoxic molecules, which may amplify local inflammatory responses and contribute to neuronal and synaptic dysfunction [33].

Dendritic cells (DCs) are scarce in the healthy CNS, but can accumulate in meningeal and choroid plexus regions in pathological conditions. Here, DCs sample CNS-derived antigens and present them to T cells, thereby initiating or sustaining adaptive immune responses [34]. Through the modulation of cytokine profiles in meningeal and perivascular spaces, DCs can indirectly influence synaptic plasticity and neuronal function [35].

Finally, increased numbers of B cells have been detected in the cerebrospinal fluid (CSF), brain parenchyma, and perivascular spaces during CNS inflammation, underscoring their contribution to neuroimmune responses. Beyond antibody production, B cells can secrete cytokines, present antigens to T cells, and modulate glial activation, thereby influencing synaptic function in disorders such as multiple sclerosis and autoimmune encephalitis [36,37].

Overall, while peripheral immune cells are not constitutive components of the CNS, their conditional recruitment during disease states can significantly shape the neuroinflammatory milieu. Through direct and indirect interactions with resident glial cells, infiltrating immune populations can amplify inflammatory signaling and lower the threshold for synaptic dysfunction, particularly when immune activation becomes chronic or unresolved [38,39].

### 2.4. Cytokines and Chemokines

Cytokines and chemokines are key soluble mediators that orchestrate immune communication and inflammatory signaling within the CNS, where they are produced mainly by microglia and astrocytes in response to injury, infection, or perturbations of tissue homeostasis [40].

Upon binding to their cognate receptors, cytokines activate intracellular signaling pathways, including JAK–STAT and MAPK cascades, which regulate innate immune responses and shape adaptive immunity [41]. These signaling events promote immune cell recruitment and can establish feed-forward inflammatory loops, when not properly regulated. Sustained or dysregulated cytokine production can amplify glial activation, enhance oxidative stress, and interfere with neuronal and synaptic homeostasis. Several cytokines have been consistently implicated in neuroinflammatory processes, including IL-1β, IL-6, TNF-α, IFN-γ, IL-23, and granulocyte–macrophage colony-stimulating factor (GM-CSF) [42]. Importantly, the effects of cytokines on CNS function are highly dependent on their concentration, temporal dynamics, and cellular context.

Chemokines, a large family of chemotactic cytokines including CCL2, CXCL1, and CCL5, also play important immunomodulatory roles within the CNS. Beyond their classical role in directing leukocyte migration, chemokines can modulate neuronal excitability, synaptic transmission, and circuit connectivity [43,44]. However, the precise mechanisms through which chemokines influence synaptic plasticity, and whether these effects are direct or mediated by glial signaling, remain incompletely understood and represent an area of active investigation.

### 2.5. Blood-Brain Barrier

Within the neurovascular unit, the interaction among pericytes, endothelial cells, astrocytes, and neurons is critical for the structural and functional maintenance of the BBB [45]. Uncontrolled or chronic neuroinflammation, involving sustained activation of microglia and astrocytes, can compromise BBB integrity, leading to increased permeability to peripheral immune cells, particularly lymphocytes [18]. This increased permeability facilitates immune cell infiltration and cytokine diffusion into the CNS, thereby amplifying local inflammatory signaling and contributing to neuronal and synaptic impairment. Importantly, BBB dysfunction should not be viewed solely as a passive consequence of inflammation, but rather as an active modulator of neuroimmune interactions that shapes the magnitude, timing, and persistence of inflammatory responses within the CNS.

## 3. Immune Modulation of Neuroplasticity

The term “neuroplasticity” comprises a series of functional and structural mechanisms that lead to neuronal remodeling, formation of novel synapses and neurogenesis. It expresses the ability of neural circuits to adapt their structure and function in response to experience and environmental demands [46]. Immune signaling plays a modulatory and context-dependent role in neuroplasticity. Under physiological conditions, immune mediators contribute to synaptic stability and adaptive plasticity, as shown in several brain regions, including the hippocampus, nucleus accumbens, and striatum. Conversely, disruption of brain homeostasis accompanied by sustained activation of microglia and astrocytes can interfere with synaptic plasticity and circuit function. Cytokines, through which immune cells, neurons and glia communicate, exert an effect on neuroplasticity that is highly context-dependent. At low and tightly regulated concentrations, cytokines such as IL-1β, IL-6, and TNF-α participate in synaptic scaling, LTP, and neurogenesis [47,48]. In contrast, excessive or chronic immune activation, triggered by infection, injury, or prolonged stress, leads to high cytokine levels that disrupt synaptic homeostasis. This dysregulation impairs LTP, reduces neurotrophic support (e.g., brain-derived neurotrophic factor, BDNF), and promotes structural and functional synaptic deficits, which are associated with neurodegenerative and neuropsychiatric disorders [49]. Importantly, whether immune dysregulation directly drives synaptic failure or acts as an amplifier of ongoing circuit instability remains dependent on disease context and temporal dynamics.

Microglia are crucial for regulating neurogenesis, affecting the proliferation and survival of neural precursor cells [50]. During development, microglia promote synaptogenesis and circuit refinement through secretion of trophic factors (e.g., BDNF, IGF-1) [51,52]. They continually survey the microenvironment, sculpt synapses and promote synaptic plasticity in an activity-dependent manner, allowing for the remodeling of brain circuits and contributing to the maintenance of the excitation-inhibition balance at the network level [50]. Indeed, mice with a loss-of-function mutation in DAP12, a transmembrane polypeptide selectively expressed in microglia and some oligodendrocytes, display prenatal microglia deficiency that leads to synaptic alterations in adulthood [53,54]. The mechanism underlying synaptic dysfunction is likely an inflammatory state, since these mice overexpress many genes coding for inflammatory proteins, including IL-1β, IL-6 and nitric oxide synthase 2 (NOS2).

Both microglia and astrocytes are involved in synaptic pruning, a selective elimination of inappropriate/unnecessary synapses and axonal branches during development and in the adult brain, that is necessary for shaping local neural circuits [51,55]. The precise molecular mechanisms behind synaptic pruning have not been completely understood yet, although the involvement of both activation of the classical complement pathway for microglia [56] and of phagocytic receptors Multiple EGF-like-domains 10 (MEGF10) and MER Tyrosine Kinase (MERTK) for astrocytes [57] have been hypothesized.

In the postnatal period, astrocytes give a major contribution to synaptogenesis through the secretion of neurotrophic factors like epidermal growth factor (EGF) and BDNF [58,59]; in fact, synaptogenesis and astrogenesis occur simultaneously [60]. Astrocytes mainly support the formation of glutamatergic synapses, but also inhibitory synapses, by secreting synaptogenic proteins like thrombospondins (TSPs) and hevin (also known as SPARC-like1, secreted protein acidic and rich in cysteine) [61,62]. Moreover, astrocytes have been implicated in the regulation of oligodendrocyte development and in the formation of myelin through the release of substances like Platelet-Derived Growth Factor (PDGF) and Fibroblast Growth Factor (FGF) [63], as well as in the phagocytosis of myelin debris and dead cells [64].

The role of astrocytes in synaptic transmission is also well-represented in the so-called “tripartite synapse”, a functional and physical unit that allows a bidirectional interaction between synapses and astrocytes. It is formed by the pre- and post-synaptic terminals of two neurons and an astrocyte, which intimately wraps around the other two components [65]. Here, astrocytes release gliotransmitters, such as D-serine, glutamate and ATP, in response to local synaptic activity, contributing to modulate synaptic transmission [66]. Moreover, astrocytes modulate synaptic activity by providing neurons with neurotransmitter substrates, and by means of GLT-1 and GLAST transporter-mediated reuptake of glutamate from the synaptic cleft [67], hence additionally protecting neurons from excitotoxicity. They also support neuronal survival through the scavenging of extracellular ROS [58,59]. Under physiological conditions, astrocytes actively participate in the metabolic clearance of amyloid beta (Aβ). Specifically, the receptor RAGE expressed on the astrocytic membrane binds Aβ, facilitating its phagocytosis and subsequent lysosomal degradation to maintain Aβ homeostasis [22].

Astrocytes exhibit variations in intracellular calcium ion (Ca^2+^) concentration, which occur both spontaneously and in response to neurotransmitter release from neighboring synaptic connections [68]. Moreover, they regulate potassium (K^+^) buffering and lactate shuttling to neurons via monocarboxylate transporters (MCTs), a process associated with LTP and memory formation [69]. Notably, inflammatory astrocytes show impaired K^+^/glutamate buffering, altered Ca^2+^ signaling, and disrupted gap-junction coupling, all contributing to neuronal hyperexcitability and synaptic instability [70].

Therefore, astroglia play a dominant role in the maintenance of adaptive homeostasis of the CNS, a concept closely linked to that of brain maintenance as part of cognitive reserve [71].

### 3.1. Hippocampus

The immune system contributes to the regulation of hippocampal neurogenesis and plasticity, representing the basis of learning and memory processes. In particular, experimental evidence in animal models indicates a selective contribution of systemic CD4+ T cells. Indeed, immune-deficient mice with systemic depletion of CD4+ T lymphocytes showed reduced hippocampal neurogenesis, altered reversal learning in the Morris water maze, and decreased BDNF expression in the brain [72]. On the other hand, reconstitution with CD4+, but not CD8+, T cells restored hippocampal neurogenesis and cognitive performance [72], highlighting a lymphocyte population-selective role. Notably, CNS-specific CD4+ T cells were shown to accumulate in the meninges during cognitive tasks, where they release IL-4, promote a trophic meningeal environment, and induce BDNF production by resident cells [73]. It has been proposed that CNS-specific CD4+ T cells are primed by antigen-presenting cells that sample CNS-derived antigens, generating memory T cells that populate meningeal and CSF compartments [74]. These macrophages phagocytize and process self-antigens from the CNS, such as myelin or neural debris, presenting them and stimulating naive T cells in the periphery, leading to the formation of CNS-specific memory T cells. These memory cells eventually migrate to the meningeal CSF, where they can be reactivated by brain-surveying macrophages, prompting the release of neuroprotective cytokines, like IL-4 and TGF-β, and neurotrophic factors, like BDNF, that contribute to healthy cognitive function, learning and memory [74].

Infiltrating macrophages are also crucial for maintaining brain homeostasis. Together with glial cells, they clear dead cells and debris, neutralize toxic compounds, produce growth factors essential for cell survival and renewal, and down-regulate pro-inflammatory factors like IL-1β and TNF, contributing to regulate the brain’s physiological environment [75]. Physiological levels of TNF-α play essential roles in hippocampal synaptic plasticity, regulating homeostatic synaptic scaling and supporting LTP induction thresholds [76]. TNF released by microglia regulates homeostatic synaptic scaling, up-regulates the number of α-amino-3-hydroxy-5-methyl-4-isoxazolepropionic acid receptors (AMPARs) at the post-synaptic level in response to decreased neuronal activity, allowing the homeostatic adjustment of neuronal excitability, and supports the induction threshold of LTP [76]. In contrast, sustained TNF-α elevation impairs hippocampal LTP, disrupts glutamatergic homeostasis, and contributes to cognitive decline, as observed in models of neurodegeneration, chronic stress, and infection [76,77]. Similarly, low concentrations of IL-1β support memory consolidation, partly through astrocyte-mediated BDNF production [78]. Conversely, elevated IL-1β levels inhibit LTP in hippocampal subfields, alter the N-methyl-D-aspartate receptor ( NMDAR)--mediated calcium signaling, and suppress BDNF–TrkB pathways, leading to synaptic dysfunction [79,80]. Together, these findings support a model in which immune mediators fine-tune hippocampal plasticity under physiological conditions, but become detrimental when chronically dysregulated, acting primarily as amplifiers of synaptic vulnerability rather than as primary initiators of dysfunction.

### 3.2. Striatum

Immune signaling also modulates synaptic transmission and plasticity within the striatum, although the underlying mechanisms remain less well characterized than in the hippocampus [81]. Therefore, the role played by immune molecules and glial cells in the modulation of intra-striatal connections and basal ganglia activity still requires further elucidation. In fact, astrocytes exhibit region-specific molecular and functional properties, which shape local network dynamics [82,83]. Indeed, hippocampal and striatal astrocytes exhibit significant differences in gene expression pattern [83], as well as in their morphology, since striatal, with respect to hippocampal, astrocytes display larger spatial domains and contact a greater number of neurons [84]. In particular, each striatal astrocyte is estimated to contact about 11 medium spiny projection neurons (MSNs) [85], and to interact with approximately 50,700 excitatory synapses [84]. Despite their homogeneous distribution, striatal astrocytes could exhibit diverse activation patterns, thereby modulating the coordinated activity of direct and indirect pathways [86]. Through these interactions, striatal astrocytes regulate corticostriatal LTD and spike-timing-dependent plasticity, by modulating glutamate uptake and gliotransmitter release [87,88]. In an interesting study, Nagai and colleagues showed that MSNs triggered neighboring astrocytes via dendritic GABA release acting on astrocyte GABAB receptors; this resulted in up-regulation of the synaptogenic cue thrombospondin-1 (TSP1), increased excitatory synapses, enhanced corticostriatal synaptic transmission, and increased MSN action potential firing, which correlated with acute behavioral hyperactivity and disrupted attention in mice [89].

Microglial contributions to striatal synaptic regulation still remain incompletely understood, but emerging evidence indicates region-specific phenotypes that influence synaptic remodeling [90]. An interesting study hypothesized that an immune-mediated shaping of striatal synapses could be implicated in behavioural changes in an age-related way. The Authors found that, in adolescent male rats, D1-like receptors (D1Rs) in the nucleus accumbens (NAc) were downregulated and eliminated through complement-mediated microglial phagocytic activity, which correlated with social play behaviour [91]. In a study conducted in the NAc of TLR4 knockout mice, microglial TLR4 was shown to modulate NMDAR-dependent LTD, with potential impact on drug-related behaviour [92].

As shown in the hippocampus, IL-1β and TNF-α exert concentration-dependent effects in the striatum, where they have been associated with LTP (Hebbian synaptic plasticity) underlying learning and memory, and synaptic scaling (homeostatic plasticity), respectively [93]. At physiological levels, these cytokines support synaptic plasticity and homeostatic scaling, whereas chronic elevation impairs LTP/LTD induction, increases network noise, and promotes dendritic spine loss [93,94]. Notably, elevated TNF-α has been consistently reported in patients and animal models of basal ganglia disorders, including Parkinson’s disease, Huntington’s disease, and dystonia [95,96]. Regarding IL-1β, at physiological levels, this cytokine participates in the dopaminergic modulation of corticostriatal plasticity; however, pathological IL-1β signaling impairs cholinergic interneuron function, alters dopamine–glutamate balance, and disrupts the induction of LTP/LTD, contributing to motor and cognitive deficits [93].

These observations suggest that immune dysregulation in the striatum lowers the threshold for synaptic dysfunction and contributes to circuit instability in basal ganglia disorders, rather than acting as an isolated pathogenic mechanism.

Although substantial progress has been made in defining immune-mediated modulation of synaptic plasticity, several key questions remain unresolved. In particular, it is still unclear how distinct inflammatory signals converge at the synapse to produce adaptive versus maladaptive outcomes, and which cellular sources of cytokines are most relevant at different disease stages. Moreover, the extent to which immune-driven synaptic alterations represent reversible functional impairments or irreversible degenerative processes remains debated. Addressing these issues will require longitudinal studies, cell-type-specific approaches, and integrated analyses of immune, synaptic, and behavioral changes along disease progression.

## 4. Neuroinflammation and Synaptic Plasticity in Brain Conditions

**Figure 2:**
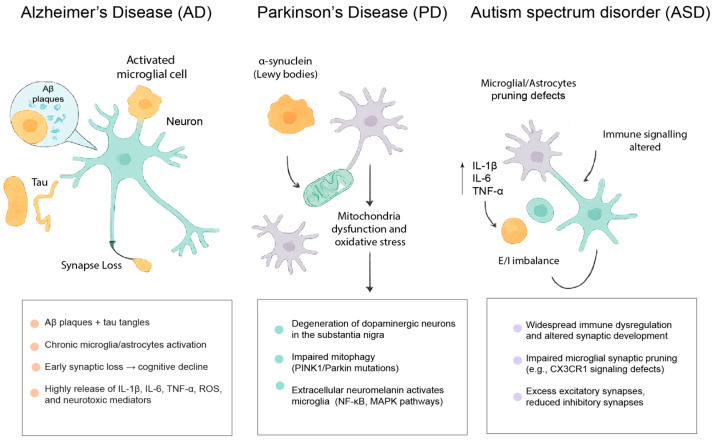
Distinct neuroimmune alterations across neurological disorders. In Alzheimer’s disease, Aβ plaques and tau pathology drive chronic microglial and astrocyte activation, leading to pro-inflammatory cytokine release, oxidative stress, and synapse loss. In Parkinson’s disease, α-synuclein aggregates and impaired mitophagy contribute to mitochondrial dysfunction and microglial activation. In neurodevelopmental and psychiatric disorders, disrupted microglial–astrocytic signaling and defective synaptic pruning result in immune dysregulation and excitatory/inhibitory imbalance.

Numerous reports described aberrant microglial and astrocytic activation in diverse neurodegenerative and neuropsychiatric conditions, including Alzheimer’s disease (AD), Parkinson’s disease (PD), amyotrophic lateral sclerosis, traumatic brain injury, schizophrenia, and autism spectrum disorders (ASD) [8,97,98]. Activation of glial cells contributes to neuroinflammation and induces pathological synaptic pruning, impaired plasticity, and altered neurotransmission. The interaction between neuroinflammation and synaptic plasticity plays a pivotal role in the pathogenesis of many brain disorders, even those for which the primary etiology is not inflammatory. In this section, we will focus on two neurodegenerative disorders (AD and PD) and on neurodevelopmental ASD (Figure 2). While a dysregulated neuroimmune signaling represents a recurring feature across AD, PD and ASD, the timing, cellular drivers, and functional consequences of inflammation differ substantially among these conditions. In neurodegenerative disorders, immune activation predominantly acts as a disease-contributing process that exacerbates synaptic vulnerability in the context of protein aggregation and neuronal loss, whereas in neurodevelopmental disorders immune perturbations may interfere with circuit maturation and synaptic pruning during critical developmental windows.

### 4.1. Alzheimer’s Disease

AD is the most prevalent neurodegenerative disorder worldwide, predominantly affecting older adults. As life expectancy improves globally, the total number of people living with dementia globally is projected to nearly triple from the current figures (around 55 million) to approximately 139 million by 2050 [99]. The clinical presentation typically begins with memory impairment (episodic memory deficits); as the disease progresses, the neurodegeneration spreads, leading to a generalized cognitive decline that subsequently affects other essential functions, including behavior, language, and visuospatial orientation, significantly disrupting daily life activities and diminishing the quality of life. In the final stages, the pathology ultimately involves the motor system [100], although some clinical evidence suggests that balance and gait impairments could be early indicators of cognitive decline [101]. The neuropathological hallmark features of AD are defined by the presence of extracellular Aβ plaques and intracellular neurofibrillary tangles (NFTs) formed by hyperphosphorylated tau, accompanied by significant synaptic and neuronal loss [102].

The initial, prevailing view of AD pathology was neuron-centric, attributing Aβ plaque deposition and NFT formation primarily to intrinsic neuronal dysfunction. This view has fundamentally changed over the decades, thanks to some research that contributed to the understanding that CNS immune response had a crucial role in the disease’s pathology. In 1975, immune-related proteins were first identified within senile plaques of AD patients, suggesting that immune responses may actively participate in disease mechanisms rather than representing purely secondary phenomena [103,104]. The 1980s brought significant histological evidence, identifying the presence of activated microglia and reactive astrocytes in and around the characteristic senile plaques of AD [105,106]. Nevertheless, inflammation was still largely interpreted as a secondary response to Aβ aggregation rather than as an active disease-contributing process. In the 1990s, however, there was a renewed interest in the neuroinflammation hypothesis, thanks to the observation that individuals who regularly took non-steroidal anti-inflammatory drugs showed up to 50% reduction in risk of developing AD compared to the general population [107]. These epidemiological findings suggested that inflammatory pathways may modulate disease risk and progression, although they did not establish a primary etiological role for inflammation. Clear evidence of neuroinflammation is documented at the cerebral level in humans affected by AD, as visualized by in vivo PET imaging [108]. Elevated levels of IL-1β and TNF-α have been detected in the CSF of AD patients and correlate with progressive neuronal dysfunction and synaptic loss [96]. The neutrophil-to-lymphocyte ratio (NLR) and C-reactive protein (CRP) have been identified as biomarkers that correlate with disease severity and faster progression of AD [109]. Moreover, emerging peripheral biomarkers, like soluble triggering receptor expressed on myeloid cells 2 (sTREM2) associated to microglial activation [110], or chitinase-3-like protein 1 (YKL-40), linked to astrocyte reactivity [111], represent a potential tool for monitoring neuroinflammation in AD.

Genetic studies further support the involvement of immune-related pathways in AD, with the identification of variants in genes like *APOE* and *TREM2*, which are involved in the inflammatory response through microglial activation [112], despite these genetic findings do not establish a clear etiological role of neuroinflammation in AD.

Neuroinflammation, particularly involving microglia and astrocytes, is now recognized as a key contributing factor of AD pathophysiology. The activation of neuroglia prompts the release of inflammatory factors, mainly cytokines and chemokines, which subsequently congregate around senile plaques and damaged neurons in the brain [113].

Aβ oligomers have an intrinsic tendency to aggregate into plaques, a process that initially triggers microglial activation. Microglia become activated via receptors like TLRs and RAGE, and produce substances that promote neuronal survival and aid in waste product removal, attempting to degrade Aβ deposits/oligomers and limiting damage. However, when clearance mechanisms become inefficient, microglia transition into a state of chronic activation that amplifies inflammatory signaling and exacerbates amyloid pathology. This persistent, dysfunctional activation leads to upregulation of AβPP expression, release of toxic ions such as Zn^+^, sustained secretion of pro-inflammatory cytokines (e.g., IL-6, TNF-α), ROS and other neurotoxic substances, creating a self-perpetuating cycle of neuroinflammation and neurodegeneration [109,114,115].

Prolonged microglial activation represents a mechanistic link between amyloid pathology and tau dysfunction. Sustained neuroinflammation with the continuous release of cytokines activates various kinases, such as glycogen synthase kinase-3β (GSK-3β), which drives tau hyperphosphorylation and formation of NFTs [116]. NFTs accumulate within axonal fibers, causing a destabilization of neuronal transport and structure, and exacerbating synaptic loss and neuronal dysfunction. The same stressed and dying neurons release molecules, such as DAMPs and S100β, that further activate glial cells [117].

Astrocytes also participate in these pathogenic mechanisms and become reactive in response to Aβ and tau pathology, undergoing isomorphic, non-proliferative gliosis. Initially, these reactive astrocytes, along with reactive microglia, assume a protective role since they constitute a barrier surrounding senile plaques and limiting neuronal damage [118]. As AD progresses, sustained astrocyte reactivity becomes maladaptive. In the advanced stages of the disease, “glial paralysis” occurs since these cells become less functional, which facilitates neuronal death and the translation into clinical dementia [119,120,121,122]. Upon activation, astrocytes secrete pro-inflammatory cytokines, chemokines and ROS that sustain a pro-inflammatory environment [123]. Of note, reactive astrocytes express components like AβPP, β-secretase and γ-secretase, thus contributing to amyloid production in the brain [123].

In AD, pro-inflammatory cytokines, such as IL-1β, IL-6, TNF-α and TGF-β, are elevated, particularly close to Aβ plaques, contributing to create a chronic neuroinflammatory environment [101]. TNF-α-mediated BBB alterations may facilitate peripheral immune cell infiltration and impair Aβ clearance, thereby amplifying local inflammation and amyloid accumulation [124]. Moreover, together with IL-1β, it stimulates NFTs formation. IL-6 exerts detrimental effects on neuronal communication, being responsible for synaptic dysfunction and cognitive decline [125]. With AD progression, there is a shift towards anti-inflammatory cytokines, such as IL-10, as a compensatory mechanism to mitigate neurotoxicity; however, their effects are not sufficient to counteract the progression of the inflammatory process.

Among neuroinflammatory mechanisms, oxidative stress plays a central role in synaptic dysfunction [126]. Oxidative stress is primarily induced by ROS production enhanced both by both neuroglial activation and mitochondrial dysfunction [127]. Excessive ROS, along with Aβ and tau, can lead to NMDARs dysfunction and internalization, impairing synaptic plasticity [128]. The inflammatory response with microglia activation triggered by Aβ plaques intensifies oxidative damage in a destructive loop that accelerates synaptic impairment. Furthermore, ROS and reactive nitrogen species activate pathways that lead to tau hyperphosphorylation, again driving synaptic dysfunction [129].

Synapses represent a vulnerable and critical target in AD, and synaptic loss is considered the main biological correlate of cognitive decline. Experimental studies show that synaptic dysfunction is an early and primary target of Aβ toxicity in AD, preceding neuronal death [130].

Soluble Aβ oligomers, rather than fibrillar plaques, consistently impair NMDAR-dependent LTP and facilitate LTD in hippocampal preparations, shifting plasticity toward synaptic weakening [131,132,133]. These oligomers disrupt glutamatergic signaling by overactivating GluN2B-NMDARs and Metabotropic Glutamate Receptor subtype 5 (mGluR5), increasing extrasynaptic glutamate and Ca^2+^ influx, and activating pathways (e.g., calcineurin, NF-κB) that drive spine retraction and AMPARs internalization [134,135,136].

Neuroinflammation amplifies this synaptic vulnerability. Cytokines such as TNF-α and IL-1β alter synaptic scaling, suppress LTP, promote LTD, and increase AβPP expression, sustaining a cycle of Aβ production and synaptic impairment [137,138]. Complement pathways also become aberrantly reactivated: C1q and C3 tag synapses for microglial engulfment, leading to pathological synapse loss [139,140,141].

Human data align with experimental findings. Synaptic vesicle glycoprotein 2A (SV2A)-PET imaging reveals early reductions in synaptic density in hippocampus and posterior cingulate, correlating with memory impairment and tau burden [142,143,144]. CSF synaptic biomarkers like neurogranin, synaptosomal-associated protein 25 (SNAP-25) and GAP-43 are elevated in prodromal and clinical AD and predict progression to dementia [145,146,147,148].

Collectively, these observations support a model in which immune-mediated signaling represents a central modulatory axis connecting amyloid pathology, tau dysfunction, oxidative stress, and synaptic vulnerability in a stage-dependent manner [122].

Overall, current evidence supports a model in which neuroinflammation does not initiate AD pathology, but instead interacts with amyloid and tau cascades to accelerate progressive synaptic dysfunction and cognitive decline. Consistent with this view, the limited efficacy of broad anti-inflammatory therapies in clinical trials highlights the need for temporally and mechanistically targeted interventions.

### 4.2. Parkinson’s Disease

PD is the second most common neurodegenerative disorder after AD, with a prevalence of over 7 million people worldwide in 2020 [149]. Its cardinal motor features (bradykinesia, resting tremor, rigidity, and loss of postural reflexes) result from the progressive loss of midbrain dopaminergic (DA) neurons in the substantia nigra (SN) and the appearance of Lewy bodies (LBs), α-synuclein-rich intracellular inclusions [150]. PD has come to be viewed over time as not just a pure movement disorder, but a heterogeneous disease with a constellation of clinically significant non-motor symptoms. It has similarly been observed that its pathology involves extensive regions of the nervous system, diverse neurotransmitters, and protein aggregates other than LBs [151].

The precise molecular mechanisms underlying PD pathogenesis remain incompletely understood, although extensive research has generated multiple hypotheses involving protein aggregation, mitochondrial dysfunction, oxidative stress, and prion-like transmission [126,152,153]. These mechanisms converge on inflammatory and immune-related pathways, positioning neuroinflammation as a key component of PD pathophysiology rather than as an initiating trigger. Studies in adult mice demonstrate that the SN has the highest density of resting microglia within the CNS, constituting about 12% of the total cell population in this region [154]. Activation of microglia leads to the release of pro-inflammatory cytokines that can induce reactive astrocyte phenotypes, contributing to synaptic dysfunction and neuronal vulnerability. The relatively low astrocyte-to-microglia ratio in the SN has been proposed as a factor contributing to the selective vulnerability of DA neurons, limiting astrocytic buffering capacity against oxidative stress, protein aggregation, and inflammatory mediators [155]. A further factor contributing to the vulnerability of SN in PD is the presence of high concentrations of neuromelanin. Neuromelanin is the pigmented by-product resulting from catecholamine oxidation. It plays a neuroprotective role by serving as the main storage molecule for iron and other metals within the DA neurons of the SN [156]. During DA neuron degeneration, extracellular neuromelanin activates surrounding microglia, triggering pro-inflammatory signaling pathways such as NF-κB and MAPK and promoting neuromelanin phagocytosis [157]. Neuromelanin-associated iron release, reacting with hydrogen peroxide generated during dopamine metabolism, enhances ROS production [158], thereby amplifying oxidative stress and neurotoxicity in the PD brain.

Most of the immune species express dopaminergic receptors and the required machinery for the synthesis, metabolism, and storage of dopamine [159]. It has been observed that, under physiological conditions in the nigrostriatal pathway, the high dopamine concentration stimulates low-affinity receptors (D1Rs and D2Rs), thereby exerting anti-inflammatory effects [160]. Conversely, dopamine depletion in PD leads to preferential activation of high-affinity receptors (notably D3Rs), promoting pro-inflammatory signaling and contributing to sustained neuroinflammation and neuronal vulnerability [159]. Numerous clinical and pre-clinical studies have demonstrated the burden of neuroinflammation in PD, including increased levels of pro-inflammatory cytokines in blood, CSF, and brain tissue of PD patients [161,162], activated microglia in PD brains [163,164], and brain invasion of CD8+ and CD4+ T lymphocytes [165]. Furthermore, neuroimaging studies using radiotracers specific to activated microglia confirmed neuroinflammation in PD [166]. About the role of astroglia in PD-associated neuroinflammation, studies yield contradictory results. Some evidence on human PD tissues have shown null or mild increases of astrocytes and/or GFAP immunoreactivity [167,168], while neurotoxin-based PD animal models have displayed significant astrogliosis [169,170]. These discrepancies likely reflect differences in disease stage, brain region, and experimental model, underscoring the context-dependent contribution of astrocytes to PD pathology.

It is assumed that PD pathogenesis involves a complex interplay between genetic susceptibility, environmental factors, and aging [171,172]. Many genetic and environmental risk factors converge on pathways regulating immune and inflammatory responses, leading to destabilization of neuronal homeostasis and influencing disease risk and progression.

**Environmental triggers of inflammation in PD:** Since most PD cases are sporadic, environmental factors interacting with low-penetrance susceptibility genes are considered major contributors to disease onset. Exposure to chemical neurotoxins is known to modulate inflammatory responses in PD and has been extensively used to model disease mechanisms in animals. PD-associated neurotoxins induce DA neuronal degeneration accompanied by reactive microgliosis, oxidative stress, and mitochondrial dysfunction, thereby exacerbating neurotoxicity [173,174]. One of the most widely used neurotoxins is 1-methyl-4-phenyl-1,2,3,6-tetrahydropyridine (MPTP), whose active metabolite MPP^+^ inhibits mitochondrial complex I, reducing ATP production and increasing oxidative stress [175,176]. MPP^+^ also disrupts mitochondrial trafficking along dopaminergic axons, ultimately contributing to synaptic loss [176,177]. Notably, sustained microglial activation and extracellular neuromelanin deposition have been observed years after MPTP exposure, even in the absence of LBs [177], indicating long-lasting immune dysregulation independent of overt protein aggregation. Structural homology between MPTP and several pesticides and herbicides has supported epidemiological and experimental studies linking environmental toxins to PD. For instance, the pesticide rotenone potently inhibits mitochondrial complex I in DA neurons, triggering ROS production, ATP depletion, mitochondrial depolarization, Ca^2+^ dysregulation, glutamate-mediated excitotoxicity, and sustained inflammatory signaling [126].

Inflammation stemming from viral and bacterial infections is increasingly recognized as a risk factor for PD. Studies have shown that PD patients have an elevated infectious burden, measured by antibody titers against pathogens like cytomegalovirus, Epstein Barr virus, Borrelia burgdorferi and Chlamydophila pneumoniae, and contextually increased serum levels of SNCA, IL-1β and IL-6 [178]. LPS injected in murine models and acting primarily through TLR4 expressed by microglia, is sufficient to induce an inflammatory response in the SN that results in loss of DA neurons [179]. Together, these findings support a model in which inflammatory stimuli act as amplifiers of neuronal vulnerability, lowering the threshold for synaptic and dopaminergic circuit dysfunction rather than serving as primary disease initiators.

**Genetic inflammatory contributors in PD:** The discovery of monogenic forms of PD has substantially contributed to comprehending the underlying pathophysiology of this disorder. Although PD risk loci exert their effects through diverse mechanisms, convergence on immune and inflammatory pathways has emerged as a recurring feature influencing disease susceptibility and progression. Many PD-associated mutations are thought to modulate inflammatory responses via pathways involving oxidative stress, rather than acting as direct inflammatory triggers [126]. Furthermore, numerous PD risk loci, such as *DJ-1* and *GBA*, possess functional roles within the immune system [180], and immune cells express many of the genes involved in the genetic risk of PD [181]. Genome-wide association studies further suggest a strong involvement of both the innate and adaptive immune systems in the genetic susceptibility to PD [182]. Mutations in the leucine-rich repeat kinase 2 (*LRRK2*) gene account for about 4% of all familial cases, representing the most frequent genetic cause of PD [183]. LRRK2 is involved in multiple cellular processes, including autophagy, endolysosomal trafficking, mitochondrial function, cytoskeletal dynamics, synaptogenesis, and immune system modulation [184]. LRRK2 has been implicated in the modulation of immune responses, influencing inflammatory signaling pathways in both central and peripheral immune cells. LRRK2 regulates cytokine production through both TLR-dependent and TLR-independent mechanisms [185]. Moreover, it modulates microglial activity through signaling pathways such as NF-κB and p38 MAPK [186]. Accordingly, *LRRK2* mutations have been proposed to increase susceptibility to infection- and inflammation-driven stressors. Inhibition of LRRK2 kinase suppresses microglial activation in transgenic α-synuclein mice and attenuates microglial inflammatory responses induced by LPS exposure and α-synuclein overexpression [187]. Moreover, *LRRK2* knockout rodents show mitigated neuroinflammatory effects and exhibit partial protection against parkinsonian toxins and viral inducers of inflammation [188]. These findings support a role for LRRK2 in shaping neuroimmune responses that modulate synaptic and neuronal vulnerability in PD, rather than acting as a primary pathogenic driver.

Mutations in the *SNCA* gene, encoding α-synuclein, are associated with familial parkinsonism with variable clinical phenotypes [189]. α-synuclein is a small, soluble protein primarily localized at presynaptic terminals, mitochondria-associated endoplasmic reticulum membranes, and within the nucleus. Although its physiological role remains incompletely understood, its presynaptic localization suggests a function in regulating synaptic activity and plasticity through modulation of synaptic vesicle maturation and release [190]. Indeed, α-synuclein accumulation impairs synaptic vesicle fusion and clustering, compromising neurotransmitter release [191]. Moreover, α-synuclein oligomers exert toxic effects at synapses, impairing LTP and enhancing basal synaptic transmission in distinct neuronal populations through NMDAR-dependent mechanisms [192,193,194]. *SNCA* mutations cause α-synuclein overexpression or increased aggregation propensity, which can indirectly promote neuroinflammatory responses. Oligomeric and fibrillary α-synuclein species released into the extracellular space by stressed DA neurons act as DAMPs, activating microglia through TLR2 and inducing pro-inflammatory cytokine production [195]. Furthermore, extracellular misfolded α-synuclein can propagate from neurons to glial cells in a prion-like manner [196]. In neurons, mutant or overexpressed α-synuclein aggregates induce mitochondrial dysfunction, ROS production, and oxidative stress, thereby sustaining inflammatory signaling [190,197]. Collectively, α-synuclein represents a central node in a complex network linking protein aggregation, immune activation, and synaptic dysfunction in PD, as inflammatory mediators released downstream of α-synuclein pathology influence synaptic integrity and plasticity.

Homozygous mutations in the *GBA1* gene, encoding the lysosomal enzyme glucocerebrosidase (GCase), cause Gaucher’s disease, whereas heterozygous mutations confer increased risk for PD [198].

Studies in asymptomatic *GBA* mutation carriers reveal aberrant immune and inflammatory responses occurring early, prior to overt neurodegeneration [199]. Indeed, *GBA* is highly expressed in immune cells, including monocytes/macrophages and lymphocytes [200]. GCase activity is significantly reduced in circulating monocytes from PD patients compared with healthy controls [201]. Of note, PD patients carrying *GBA* mutations display elevated plasma levels of inflammatory cytokines, including IL-8 and macrophage inflammatory protein-1α [202]. Reduced GCase activity in microglia impairs phagocytosis of parenchymal debris and pathological α-synuclein species released by degenerating neurons. This impaired clearance promotes persistence of aggregated substrates and sustained activation of surrounding immune cells, thereby maintaining a chronic inflammatory milieu [203].

The genes *PRKN* and *PINK1*, encoding the proteins Parkin and PTEN-induced kinase 1 respectively, are central to early-onset, autosomal recessive PD [204,205]. Both proteins are key regulators of mitophagy, the selective clearance of damaged mitochondria [206]. *PRKN* and *PINK1* mutations are associated with mitochondrial dysfunction accumulation of damaged mitochondria, and increased production of ROS and mitochondrial-DAMPs [207]. These DAMPS are recognized by immune complexes, including NLRP3 (nucleotide-binding oligomerization domain leucine-rich repeat and pyrin domain-containing protein 3) inflammasome, highly expressed in microglia, thus triggering an immune response with massive release of pro-inflammatory cytokines [208]. PINK1 and Parkin are also expressed in microglia where their dysfunction impairs microglial metabolic fitness and clearance of cellular debris and aggregated proteins (like α-synuclein), thereby sustaining a chronic neuroinflammation [209]. Failure of mitophagy leads to the accumulation of dysfunctional mitochondria at the synaptic sites, resulting in excessive ROS production and insufficient ATP supply required for vesicle cycling and neurotransmitter release. PINK1 also exerts non-mitophagy functions, including phosphorylation of synaptic proteins such as Miro, regulating mitochondrial trafficking and localization at synaptic terminals [210]. The combination of energy failure, oxidative damage, and impaired mitochondrial trafficking contributes to progressive synaptic dysfunction and impaired dopamine release, which represent early and central features of PD pathophysiology [211]. Thus, the PINK1/Parkin pathway provides a mechanistic link between mitochondrial dysfunction, immune activation, and early synaptic failure in PD.

Redox imbalance disrupts trafficking and stability of glutamatergic and dopaminergic receptors, altering synaptic scaling and lowering the threshold for synaptic depression. Experimental evidence indicates that oxidative stress facilitates NMDARs overactivation and AMPARs internalization, thereby reducing synaptic efficacy [192,193]. ROS further impair synaptic vesicle dynamics by oxidizing proteins involved in vesicle docking and recycling, leading to reduced neurotransmitter release and early synaptic failure [191].

Overall, oxidative stress acts as a convergent mechanism that amplifies synaptic vulnerability in PD, reinforcing inflammatory and degenerative pathways that accelerate early circuit dysfunction rather than serving as an independent initiating event.

### 4.3. Neurodevelopmental Disorders

Microglia and astrocyte abnormalities have been found in some neurodevelopmental disorders, although it remains unclear whether these abnormalities are causative, permissive, or consequential to disease pathology [212]. Since both microglia and astrocytes play key roles in several developmental brain processes, glial deficiencies are reasonably thought to result in developmental abnormalities. Indeed, when occurring during developmentally sensitive windows, prolonged or dysregulated microglial activation can interfere with normal neurodevelopmental trajectories [213]. ASD is a complex neurodevelopmental disorder characterized by early onset of restricted or repetitive behaviors and deficits in social interaction and communication. However, its clinical presentation is highly variable, ranging from mild to severe forms. Both genetic and environmental factors are believed to contribute to ASD pathogenesis [214]. The prevalence of ASD has increased significantly over the last few decades [215]. Although the precise pathophysiological mechanisms underlying ASD remain unclear, accumulating evidence suggests that dysregulated immune signaling may contribute to disease susceptibility and phenotypic heterogeneity. Several high-confidence ASD variants converge upon inflammatory pathways [216], and, among environmental risk factors, maternal immune activation (MIA) has been implied [217].

The majority of studies report increased levels of inflammatory cytokines and chemokines, such as IL-1β, IL-6, and TNF-α [217], as well as increased numbers of innate immune circulating cells and monocyte dysregulation [218,219] in ASD compared to typically developing children. Notably, increasing cytokine levels have been associated with greater behavioral impairment [220]. Moreover, postmortem evaluation of ASD brain tissue has confirmed neuroinflammation and microglial activation [221,222]. While these findings indicate a robust association between immune alterations and ASD, they do not establish a uniform causal role across the autism spectrum.

Many studies have assessed the impact of immune changes on ASD-related behaviors using genetic and environmental animal models [214,223].

As comprehensively discussed above in this review, microglia and astrocytes influence synapse formation and function, and extensive neuron-glia signaling is required for a proper assembly of neural circuits from early stages of development. Below, we summarize key experimental evidence indicating that glial–synaptic dysfunction can contribute to altered circuit development in neurodevelopmental disorders, including ASD, Rett syndrome, and Fragile X syndrome. These studies support a role for innate immune signaling and synaptic dysfunction as interacting contributors to disease mechanisms. One proposed mechanism involves disruption of microglia-mediated synaptic pruning during postnatal development, as observed in mice lacking CX3CR1, a receptor exclusively expressed by microglia. *CX3CR1*-knockout mice show reduced microglial recruitment to synaptic regions, defective synaptic pruning, excessive spine density, impaired synaptic maturation, and weakened functional connectivity, associated with ASD-like behaviors [224,225]. Microglia also regulate critical period timing: dysfunctional microglial maturation can lead to inappropriate closure or prolongation of developmental windows, contributing to sensory hypersensitivity and social deficits observed in ASD models [226].

To investigate glial contributions to synaptic alterations in ASD, Traetta and colleagues studied primary cultures of cortical neurons, microglia and astroglia from rats prenatally exposed to valproic acid (VPA), a validated model of ASD. In the prefrontal cortex (PFC), increased excitatory synapse number was observed at postnatal day 3, followed by reactive microgliosis and astrogliosis at postnatal day 35 compared to controls. Cortical microglia and astroglia from VPA-exposed animals exhibited reactive morphology and increased pro-inflammatory cytokine expression. Interestingly, microglia isolated from VPA-exposed rats displayed altered reactivity profiles, remaining non-reactive and continuing to promote neurite outgrowth when co-cultured with VPA neurons. In microglia-astroglia co-cultures, microglia from VPA-exposed rats induced enhanced astrocyte reactivity, suggesting disrupted microglia-astrocyte crosstalk [227].

In another VPA-based ASD model, animals displayed core ASD-like behaviors together with abnormal expression of synapse-related proteins in the PFC, increased excitatory synapses, decreased inhibitory synapses and polarization of microglia towards a pro-inflammatory phenotype [228]. This study also supports a protective role for TREM2, a microglial receptor of the immunoglobulin superfamily [229]. Prenatal VPA exposure was associated with reduced *TREM2* expression in microglia [230]. Overexpression of *TREM2* in primary microglia–neuron cultures attenuated inflammatory responses and restored gephyrin expression, improving synaptic development [228].

Recently, the effect of LPS-induced MIA on neuroinflammatory changes and synaptic morphology were examined in adolescent male rat offspring. These animals exhibited elevated peripheral cytokines, microglial activation, increased pro-inflammatory cytokine expression, and oxidative stress. Structural synaptic abnormalities were accompanied by reduced presynaptic proteins and downregulation of postsynaptic scaffolding proteins [231]. Jing and colleagues replicated the VPA-induced ASD model, reporting increased locomotor activity, social impairment, repetitive behaviors, upregulation of NF-κB signaling, elevated microglial numbers and synaptic dysfunction. Acute LPS exposure in VPA-exposed rats further exacerbated ASD-like behaviors, promoting NF-κB activation, microglial overactivation, and downregulation of key synaptic proteins [232]. In a two-hit model, prenatal MIA induced by poly (I:C) was combined with hypoxia-ischemia (HI) at postnatal day 10. MIA alone produced limited microglial activation but sensitized offspring to exaggerated immune responses following a secondary insult. Neonatal HI alone did not induce autistic-like behaviors, whereas combined MIA/HI exposure resulted in synergistic effects, including autistic-like behaviors, monocytic infiltration, increased perineuronal nets, reduced synaptic density, and downregulation of ASD-associated synaptic proteins such as PSD-95 and Homer-1. Blockade of monocyte infiltration significantly attenuated behavioral abnormalities [233].

The *R451C-NLGN3* mouse model carries the ASD-liked mutation in neuroligin 3, a postsynaptic adhesion protein essential for synapse formation and maturation [234]. *R451C-NLGN3* mice exhibit deficits in social interaction and increased repetitive behaviors [235,236]. Altered microglial and astrocyte morphology consistent with a reactive state has been reported in the hippocampus [237]. Changes in synaptic protein expression, including reduced SNAP-25 levels in the cortex and increased PSD-95 levels in the striatum have also been described [236,237]. In line with these findings, significant impairments in striatal synaptic function have been reported in *R451C-NLGN3* mice [236,238].

Mutations in the X-linked methyl-CpG binding protein 2 (*MeCP2*) gene cause Rett syndrome, a rare neurodevelopmental disorder that primarily affects girls. Genetic mouse models of Rett syndrome display neurological symptoms similar to humans [239]. In vitro studies show that MeCP2-null microglia produce elevated glutamate levels with neurotoxic effects, inducing dendritic and synaptic impairment [240]. MeCP2-null astrocytes exhibit altered BDNF and cytokine production and impair dendritogenesis [241,242].

Fragile-X syndrome (FXS), caused by mutations in the fragile X mental retardation 1 (*FMR1*) gene, also involves glial dysfunction. Co-culture of healthy neurons with FXS astrocytes delays dendritic maturation and alters synaptic protein expression [243]. Microglial alterations in FXS include abnormal cytokine expression and excessive synaptic engulfment during early development, contributing to network instability and cognitive dysfunction [244].

Within this framework, immune dysregulation emerges as a modulatory factor that shapes synaptic maturation and circuit assembly in a time- and context-dependent manner, rather than as a common causal mechanism across the autism spectrum.

Collectively, these findings indicate that dysregulated glial signaling during critical developmental windows can disrupt synaptic maturation and circuit assembly. Rather than acting as a uniform causal mechanism, immune dysregulation appears to modulate disease expression and severity in a context-, time-, and genotype-dependent manner across neurodevelopmental disorders [245,246,247].

## 5. Multimodal Stimulation as a Non-Pharmacological Intervention Targeting Neuroinflammation

The emerging paradigm in treating CNS disorders emphasizes the critical importance of early intervention, acting before significant, irreversible damage occurs. This is particularly relevant for strategies aimed at modulating immune responses and limiting maladaptive neuroinflammation, which is implicated across a wide range of neurological disorders, even when inflammation is not considered the primary etiological driver [248]. Indeed, as previously discussed, neuroinflammation and dysregulation of astroglia and microglia functions can disrupt brain development and function, and, conversely, neuronal activity can influence the immune response [7].

In addition to pharmacological interventions targeting neuroinflammation, non-pharmacological approaches have gained attention as safe, accessible alternative strategies to influence the CNS environment. Among these, cognitive and physical training have been shown to exert beneficial effects in diverse neurological conditions, including cognitive impairment and dementia [249,250], PD [251,252], as well as in aging [253]. Physical exercise, for example, can attenuate neuroinflammatory signaling and promote neuroplasticity, as demonstrated in both clinical and preclinical studies [254,255,256,257].

Recently, a multimodal approach combining different types of stimulation has been proposed, as it appears to produce broader and more sustained effects compared to a unimodal interventions [258,259]. The concept of multimodal stimulation is closely linked to that of cognitive reserve. The notion of cognitive reserve, as introduced by Yaakov Stern [260], proposes that cumulative physical, cognitive and social experiences acquired throughout a life enhance brain resilience to pathology, support cerebral maintenance, and provide protection against cognitive decline in both clinical populations and older adults [261,262,263]. Within this framework, multimodal stimulation is viewed as a means to engage multiple neural systems simultaneously, potentially increasing compensatory capacity and functional adaptability.

To model multimodal stimulation in animals and investigate the impact of lifestyle-related factors on cognitive function, the paradigm of Environmental Enrichment (EE) is widely employed [264,265]. EE provides enhanced sensory, cognitive, motor, and social stimulation, recapitulating aspects of human experiences that contribute to cognitive reserve [266,267]. EE has been extensively studied as a non-pharmacological intervention in aging, psychiatric disorders, brain injuries, and neurodegenerative diseases [170,268,269]. EE engages multiple biological mechanisms, including modulation of neuroinflammatory signaling, creating a more permissive environment for neuronal function and plasticity [264,265,267,270,271]. One proposed mechanism involves normalizations of glial morphology and function. Chronic brain disorders and aging are often associated with astrocytic and microglial atrophy or functional impairment [272]. Multimodal stimulation may counteract these alterations, enhancing astrocytic support of synaptic connectivity and modulating microglial reactivity [71,273,274].

It is well established that EE promotes neurogenesis [264,275], synaptogenesis [276,277], gliogenesis [278,279], and angiogenesis [280]. These effects are associated with increased neuronal survival, enhanced dendritic arborization and spine density, and improved synaptic plasticity [277,278,281]. Accordingly, EE exposure upregulates the expression of neurotrophic factors, such as BDNF [282,283]. Notably, many of these processes are influenced by immune–glial signaling pathways, suggesting that neuroimmune modulation may contribute indirectly to EE-induced plasticity.

Focusing on PD, increasing evidence from both clinical and preclinical studies indicates that cognitive stimulation and physical training can enhance both cognitive and motor performances [284,285,286]. Although cognitive stimulation is widely implemented in PD patients, combined physical and cognitive training has been proposed as a more effective strategy to slow cognitive decline than single-modality interventions [287]. While not replacing pharmacological therapies, multimodal stimulation may complement existing treatments, potentially reducing symptom burden and enhancing quality of life in PD patients [288,289]. In the case of neurodevelopmental disorders, early therapeutic intervention is associated with improved long-term functional outcomes. Systematic reviews and meta-analyses indicate that physical activity and exercise interventions benefit communication, social interaction, stereotyped behaviors, cognitive flexibility and inhibitory control in children and adolescents with ASD [290,291]. Although the molecular mechanisms underlying these benefits are not fully defined, existing evidence implicates exercise-induced cytokines in the regulation of neuronal metabolism, neuroinflammation, and plasticity [292,293]. In ASD animal models, EE exerts beneficial effects via BDNF-related pathways, enhanced neurogenesis, and modulation of microglia-associated inflammatory responses, leading to improved synaptic plasticity and reduced ASD-like behaviors [294,295]. Early interventions combining cognitive—behavioral therapy, sensory integration, and physical activity, may improve functional outcomes in individuals with ASD [296,297]. In conclusion, further studies are required to delineate optimal timing, intensity, and modality combinations, as well as to identify the neuroimmune mechanisms mediating EE effects across heterogeneous neurological conditions.

## 6. Concluding Remarks

The present review outlined the convergence of dysregulated neuroimmune signaling and synaptic dysfunction into common pathogenic mechanisms in neurodegenerative and neurodevelopmental disorders. Across these conditions, sustained glial activation, altered cytokine signaling, and reduced availability of neurotrophic factors frequently converge to impair synaptic function and increase neuronal vulnerability. The intrinsic inflammatory response of the CNS is crucial for protection against CNS insults. A finely orchestrated and self-limiting sequence of immune events supports tissue repair and maintenance of brain homeostasis. However, in many instances, the delicate balance of the inflammatory responses can be lost, and disease may manifest. Under these conditions, neuroimmune signaling may shift from adaptive to maladaptive states. This imbalance can act as a key process in neurodegenerative disorders such as AD, where aberrant or unresolved inflammation may accelerate amyloid and tau pathology and promote early synaptic dysfunction. In other conditions, as PD, multiple pathogenic mechanisms, including mitochondrial dysfunction, protein aggregation, and oxidative stress, interact with chronic neuroinflammation establishing self-reinforcing cycles that exacerbate neuronal vulnerability and accelerate neurodegeneration. During neurodevelopment, sustained inflammatory environments may interfere with the molecular and cellular mechanisms underlying neuroplasticity, a process that is particularly critical for appropriate neural circuit formation. Considering that, for many of these neurological disorders, no effective and disease-modifying treatments are currently available, the identification of early, non-pharmacological and non-invasive interventions targeting neuroinflammation and preserving synaptic plasticity can represent a powerful complementary strategy. Among these approaches, accumulating experimental and clinical evidence supports multimodal stimulation paradigms, combining physical exercise, cognitive training, and lifestyle-related interventions, to harness potential synergistic effects. Multimodal stimulation has been associated with enhanced cognitive reserve, thereby offering a strategy to increase resilience to functional decline and delay the clinical manifestation of underlying neuropathology in neurological conditions. Such knowledge may inform the design of early, developmentally appropriate, and multimodal interventions aimed at mitigating disease severity and improving long-term outcomes.

Overall, investigation of neuroimmune crosstalk represents a rapidly evolving area of research, with important implications for understanding both physiological and pathological brain plasticity. Advancing this field will be essential for guiding the rational development of integrated pharmacological and lifestyle-based strategies tailored to disease stage, context, and individual variability.

Despite strong experimental evidence implicating neuroinflammation in synaptic dysfunction, anti-inflammatory strategies have shown limited efficacy in clinical trials. This likely reflects the dynamic and context-dependent nature of neuroimmune responses, which may exert adaptive functions at early disease stages and become maladaptive only when chronic or unresolved. In addition, neuroimmune dysregulation arises from complex and redundant signaling networks that are unlikely to be effectively targeted by single-pathway interventions. Inter-individual heterogeneity and BBB constraints further complicate therapeutic translation. Future approaches should therefore focus on temporally precise and modulatory immunotherapies, guided by immune biomarkers rather than broad immune suppression.

## Data Availability

No new data were created or analyzed in this study.

## References

[1] Dantzer R. (2018). Neuroimmune interactions: From the brain to the immune system and vice versa. Physiol. Rev..

[2] Abbott L.F., Nelson S.B. (2000). Synaptic plasticity: Taming the beast. Nat. Neurosci..

[3] Magee J.C., Grienberger C. (2020). Synaptic plasticity forms and functions. Annu. Rev. Neurosci..

[4] Bliss T.V., Lomo T. (1973). Long-lasting potentiation of synaptic transmission in the dentate area of the anaesthetized rabbit following stimulation of the perforant path. J. Physiol..

[5] Watanabe K., Kamatani D., Hishida R., Kudoh M., Shibuki K. (2007). Long-term depression induced by local tetanic stimulation in the rat auditory cortex. Brain Res..

[6] Volterra A., Meldolesi J. (2005). Astrocytes, from brain glue to communication elements: The revolution continues. Nat. Rev. Neurosci..

[7] Tian L., Rauvala H., Gahmberg C.G. (2009). Neuronal regulation of immune responses in the central nervous system. Trends Immunol..

[8] Squillace S., Salvemini D. (2022). Toll-like receptor-mediated neuroinflammation: Relevance for cognitive dysfunctions. Trends Pharmacol. Sci..

[9] Adamu A., Li S., Gao F., Xue G. (2024). The role of neuroinflammation in neurodegenerative diseases: Current understanding and future therapeutic targets. Front. Aging Neurosci..

[10] Cantero-Fortiz Y., Boada M. (2024). The role of inflammation in neurological disorders: A brief overview of multiple sclerosis, Alzheimer’s, and Parkinson’s disease’. Front. Neurol..

[11] Cohen J., Mathew A., Dourvetakis K.D., Sanchez-Guerrero E., Pangeni R.P., Gurusamy N., Aenlle K.K., Ravindran G., Twahir A., Isler D. (2024). Recent research trends in neuroinflammatory and neurodegenerative disorders. Cells.

[12] Unnisa A., Greig N.H., Kamal M.A. (2023). Modelling the Interplay Between Neuron-Glia Cell Dysfunction and Glial Therapy in Autism Spectrum Disorder. Curr. Neuropharmacol..

[13] Sharma R., Zamani A., Dill L.K., Sun M., Chu E., Robinson M.J., O’Brien T.J., Shultz S.R., Semple B.D. (2021). A systemic immune challenge to model hospital-acquired infections independently regulates immune responses after pediatric traumatic brain injury. J. Neuroinflamm..

[14] Brambilla R. (2019). Neuroinflammation, the thread connecting neurological disease: Cluster: “Neuroinflammatory mechanisms in neurodegenerative disorders”. Acta Neuropathol..

[15] Ronaldson P.T., Davis T.P. (2020). Regulation of blood-brain barrier integrity by microglia in health and disease: A therapeutic opportunity. J. Cereb. Blood Flow Metab..

[16] Waisman A., Liblau R.S., Becher B. (2015). Innate and adaptive immune responses in the CNS. Lancet Neurol..

[17] Franceschi C., Campisi J. (2014). Chronic inflammation (inflammaging) and its potential contribution to age-associated diseases. J. Gerontol. A Biol. Sci. Med. Sci..

[18] Sochocka M., Diniz B.S., Leszek J. (2017). Inflammatory response in the CNS: Friend or foe?. Mol. Neurobiol..

[19] Ransohoff R.M., Perry V.H. (2009). Microglial physiology: Unique stimuli, specialized responses. Annu. Rev. Immunol..

[20] Qin J., Ma Z., Chen X., Shu S. (2023). Microglia activation in central nervous system disorders: A review of recent mechanistic investigations and development efforts. Front. Neurol..

[21] Keren-Shaul H., Spinrad A., Weiner A., Matcovitch-Natan O., Dvir-Szternfeld R., Ulland T.K., David E., Baruch K., Lara-Astaiso D., Toth B. (2017). A Unique Microglia Type Associated with Restricting Development of Alzheimer’s Disease. Cell.

[22] Steardo L., Bronzuoli M.R., Iacomino A., Esposito G., Steardo L., Scuderi C. (2015). Does neuroinflammation turn on the flame in Alzheimer’s disease? Focus on astrocytes. Front. Neurosci..

[23] Reid J.K., Kuipers H.F. (2021). She doesn’t even go here: The role of inflammatory astrocytes in CNS disorders. Front. Cell. Neurosci..

[24] Escartin C., Galea E., Lakatos A., O’Callaghan J.P., Petzold G.C., Serrano-Pozo A., Steinhäuser C., Volterra A., Carmignoto G., Agarwal A. (2021). Reactive astrocyte nomenclature, definitions, and future directions. Nat. Neurosci..

[25] Tastan B., Heneka M.T. (2024). The impact of neuroinflammation on neuronal integrity. Immunol. Rev..

[26] Sofroniew M.V. (2015). Astrocyte barriers to neurotoxic inflammation. Nat. Rev. Neurosci..

[27] Plog B.A., Nedergaard M. (2018). The glymphatic system in central nervous system health and disease: Past, present, and future. Annu. Rev. Pathol..

[28] Satarker S., Bojja S.L., Gurram P.C., Mudgal J., Arora D., Nampoothiri M. (2022). Astrocytic glutamatergic transmission and its implications in neurodegenerative disorders. Cells.

[29] Guo Y., Zeng H., Gao C. (2021). The role of neutrophil extracellular traps in central nervous system diseases and prospects for clinical application. Oxidative Med. Cell. Longev..

[30] Ajoolabady A., Kim B., Abdulkhaliq A.A., Ren J., Bahijri S., Tuomilehto J., Borai A., Khan J., Pratico D. (2025). Dual role of microglia in neuroinflammation and neurodegenerative diseases. Neurobiol. Dis..

[31] Wattananit S., Tornero D., Graubardt N., Memanishvili T., Monni E., Tatarishvili J., Miskinyte G., Ge R., Ahlenius H., Lindvall O. (2016). Monocyte-Derived Macrophages Contribute to Spontaneous Long-Term Functional Recovery after Stroke in Mice. J. Neurosci..

[32] Abe N., Nishihara T., Yorozuya T., Tanaka J. (2020). Microglia and macrophages in the pathological central and peripheral nervous systems. Cells.

[33] Ning Z., Liu Y., Guo D., Lin W.-J., Tang Y. (2023). Natural killer cells in the central nervous system. Cell Commun. Signal..

[34] Constant O., Maarifi G., Blanchet F.P., Van de Perre P., Simonin Y., Salinas S. (2022). Role of dendritic cells in viral brain infections. Front. Immunol..

[35] Abbaoui A., Fatoba O., Yamashita T. (2023). Meningeal T cells function in the central nervous system homeostasis and neurodegenerative diseases. Front. Cell. Neurosci..

[36] Ahn J.J., Abu-Rub M., Miller R.H. (2021). B cells in neuroinflammation: New perspectives and mechanistic insights. Cells.

[37] Jain R.W., Yong V.W. (2022). B cells in central nervous system disease: Diversity, locations and pathophysiology. Nat. Rev. Immunol..

[38] Ransohoff R.M., Brown M.A. (2012). Innate immunity in the central nervous system. J. Clin. Investig..

[39] Prinz M., Priller J. (2017). The role of peripheral immune cells in the CNS in steady state and disease. Nat. Neurosci..

[40] Liddelow S.A., Guttenplan K.A., Clarke L.E., Bennett F.C., Bohlen C.J., Schirmer L., Bennett M.L., Münch A.E., Chung W.-S., Peterson T.C. (2017). Neurotoxic reactive astrocytes are induced by activated microglia. Nature.

[41] Kempuraj D., Thangavel R., Selvakumar G.P., Zaheer S., Ahmed M.E., Raikwar S.P., Zahoor H., Saeed D., Natteru P.A., Iyer S. (2017). Brain and peripheral atypical inflammatory mediators potentiate neuroinflammation and neurodegeneration. Front. Cell. Neurosci..

[42] Hamilton J.A. (2020). GM-CSF in inflammation. J. Exp. Med..

[43] Rostène W., Dansereau M.-A., Godefroy D., Van Steenwinckel J., Reaux-Le Goazigo A., Mélik-Parsadaniantz S., Apartis E., Hunot S., Beaudet N., Sarret P. (2011). Neurochemokines: A menage a trois providing new insights on the functions of chemokines in the central nervous system. J. Neurochem..

[44] Mélik-Parsadaniantz S., Rostène W. (2008). Chemokines and neuromodulation. J. Neuroimmunol..

[45] Sweeney M.D., Ayyadurai S., Zlokovic B.V. (2016). Pericytes of the neurovascular unit: Key functions and signaling pathways. Nat. Neurosci..

[46] Calabrese F., Rossetti A.C., Racagni G., Gass P., Riva M.A., Molteni R. (2014). Brain-derived neurotrophic factor: A bridge between inflammation and neuroplasticity. Front. Cell. Neurosci..

[47] Hikosaka M., Kawano T., Wada Y., Maeda T., Sakurai T., Ohtsuki G. (2022). Immune-Triggered Forms of Plasticity Across Brain Regions. Front. Cell. Neurosci..

[48] Yirmiya R., Goshen I. (2011). Immune modulation of learning, memory, neural plasticity and neurogenesis. Brain Behav. Immun..

[49] Golia M.T., Poggini S., Alboni S., Garofalo S., Ciano Albanese N., Viglione A., Ajmone-Cat M.A., St-Pierre A., Brunello N., Limatola C. (2019). Interplay between inflammation and neural plasticity: Both immune activation and suppression impair LTP and BDNF expression. Brain Behav. Immun..

[50] Dzyubenko E., Hermann D.M. (2023). Role of glia and extracellular matrix in controlling neuroplasticity in the central nervous system. Semin. Immunopathol..

[51] Parkhurst C.N., Yang G., Ninan I., Savas J.N., Yates J.R., Lafaille J.J., Hempstead B.L., Littman D.R., Gan W.-B. (2013). Microglia promote learning-dependent synapse formation through brain-derived neurotrophic factor. Cell.

[52] Lim S.-H., Park E., You B., Jung Y., Park A.-R., Park S.G., Lee J.-R. (2013). Neuronal synapse formation induced by microglia and interleukin 10. PLoS ONE.

[53] Tomasello E., Desmoulins P.O., Chemin K., Guia S., Cremer H., Ortaldo J., Love P., Kaiserlian D., Vivier E. (2000). Combined natural killer cell and dendritic cell functional deficiency in KARAP/DAP12 loss-of-function mutant mice. Immunity.

[54] Roumier A., Béchade C., Poncer J.-C., Smalla K.-H., Tomasello E., Vivier E., Gundelfinger E.D., Triller A., Bessis A. (2004). Impaired synaptic function in the microglial KARAP/DAP12-deficient mouse. J. Neurosci..

[55] Schafer D.P., Stevens B. (2013). Phagocytic glial cells: Sculpting synaptic circuits in the developing nervous system. Curr. Opin. Neurobiol..

[56] Schafer D.P., Lehrman E.K., Kautzman A.G., Koyama R., Mardinly A.R., Yamasaki R., Ransohoff R.M., Greenberg M.E., Barres B.A., Stevens B. (2012). Microglia sculpt postnatal neural circuits in an activity and complement-dependent manner. Neuron.

[57] Chung W.-S., Clarke L.E., Wang G.X., Stafford B.K., Sher A., Chakraborty C., Joung J., Foo L.C., Thompson A., Chen C. (2013). Astrocytes mediate synapse elimination through MEGF10 and MERTK pathways. Nature.

[58] Park M.H., Lee S.M., Lee J.W., Son D.J., Moon D.C., Yoon D.Y., Hong J.T. (2006). ERK-mediated production of neurotrophic factors by astrocytes promotes neuronal stem cell differentiation by erythropoietin. Biochem. Biophys. Res. Commun..

[59] Drukarch B., Schepens E., Stoof J.C., Langeveld C.H., Van Muiswinkel F.L. (1998). Astrocyte-enhanced neuronal survival is mediated by scavenging of extracellular reactive oxygen species. Free Radic. Biol. Med..

[60] Reemst K., Noctor S.C., Lucassen P.J., Hol E.M. (2016). The Indispensable Roles of Microglia and Astrocytes during Brain Development. Front. Hum. Neurosci..

[61] Elmariah S.B., Oh E.J., Hughes E.G., Balice-Gordon R.J. (2005). Astrocytes regulate inhibitory synapse formation via Trk-mediated modulation of postsynaptic GABAA receptors. J. Neurosci..

[62] Allen N.J. (2013). Role of glia in developmental synapse formation. Curr. Opin. Neurobiol..

[63] Hu X., Yu G., Liao X., Xiao L. (2023). Interactions between astrocytes and oligodendroglia in myelin development and related brain diseases. Neurosci. Bull..

[64] Li X., Ding Z., Liu K., Wang Q., Song L., Chai Z., Yu J., Ma D., Xiao B., Ma C. (2023). Astrocytic phagocytosis of myelin debris and reactive characteristics in vivo and in vitro. Biol. Cell.

[65] Perea G., Navarrete M., Araque A. (2009). Tripartite synapses: Astrocytes process and control synaptic information. Trends Neurosci..

[66] Halassa M.M., Fellin T., Haydon P.G. (2007). The tripartite synapse: Roles for gliotransmission in health and disease. Trends Mol. Med..

[67] Ullian E.M., Sapperstein S.K., Christopherson K.S., Barres B.A. (2001). Control of synapse number by glia. Science.

[68] Charles A.C., Merrill J.E., Dirksen E.R., Sanderson M.J. (1991). Intercellular signaling in glial cells: Calcium waves and oscillations in response to mechanical stimulation and glutamate. Neuron.

[69] Suzuki A., Stern S.A., Bozdagi O., Huntley G.W., Walker R.H., Magistretti P.J., Alberini C.M. (2011). Astrocyte-neuron lactate transport is required for long-term memory formation. Cell.

[70] Verkhratsky A., Nedergaard M. (2018). Physiology of Astroglia. Physiol. Rev..

[71] Verkhratsky A., Zorec R. (2024). Neuroglia in cognitive reserve. Mol. Psychiatry.

[72] Wolf S.A., Steiner B., Wengner A., Lipp M., Kammertoens T., Kempermann G. (2009). Adaptive peripheral immune response increases proliferation of neural precursor cells in the adult hippocampus. FASEB J..

[73] Derecki N.C., Cardani A.N., Yang C.H., Quinnies K.M., Crihfield A., Lynch K.R., Kipnis J. (2010). Regulation of learning and memory by meningeal immunity: A key role for IL-4. J. Exp. Med..

[74] Ron-Harel N., Cardon M., Schwartz M. (2011). Brain homeostasis is maintained by “danger” signals stimulating a supportive immune response within the brain’s borders. Brain Behav. Immun..

[75] Shechter R., London A., Varol C., Raposo C., Cusimano M., Yovel G., Rolls A., Mack M., Pluchino S., Martino G. (2009). Infiltrating blood-derived macrophages are vital cells playing an anti-inflammatory role in recovery from spinal cord injury in mice. PLoS Med..

[76] Stellwagen D., Malenka R.C. (2006). Synaptic scaling mediated by glial TNF-alpha. Nature.

[77] Liu Y., Zhou L.-J., Wang J., Li D., Ren W.-J., Peng J., Wei X., Xu T., Xin W.-J., Pang R.-P. (2017). TNF-α differentially regulates synaptic plasticity in the hippocampus and spinal cord by microglia-dependent mechanisms after peripheral nerve injury. J. Neurosci..

[78] Goshen I., Yirmiya R. (2009). Interleukin-1 (IL-1): A central regulator of stress responses. Front. Neuroendocrinol..

[79] Viviani B., Bartesaghi S., Gardoni F., Vezzani A., Behrens M.M., Bartfai T., Binaglia M., Corsini E., Di Luca M., Galli C.L. (2003). Interleukin-1β Enhances NMDA Receptor-Mediated Intracellular Calcium Increase through Activation of the Src Family of Kinases. J. Neurosci..

[80] Tong L., Prieto G.A., Kramár E.A., Smith E.D., Cribbs D.H., Lynch G., Cotman C.W. (2012). Brain-derived neurotrophic factor-dependent synaptic plasticity is suppressed by interleukin-1β via p38 mitogen-activated protein kinase. J. Neurosci..

[81] Mancini A., Ghiglieri V., Parnetti L., Calabresi P., Di Filippo M. (2021). Neuro-Immune Cross-Talk in the Striatum: From Basal Ganglia Physiology to Circuit Dysfunction. Front. Immunol..

[82] Ben Haim L., Rowitch D.H. (2017). Functional diversity of astrocytes in neural circuit regulation. Nat. Rev. Neurosci..

[83] Khakh B.S. (2019). Astrocyte-Neuron Interactions in the Striatum: Insights on Identity, Form, and Function. Trends Neurosci..

[84] Chai H., Diaz-Castro B., Shigetomi E., Monte E., Octeau J.C., Yu X., Cohn W., Rajendran P.S., Vondriska T.M., Whitelegge J.P. (2017). Neural Circuit-Specialized Astrocytes: Transcriptomic, Proteomic, Morphological, and Functional Evidence. Neuron.

[85] Octeau J.C., Chai H., Jiang R., Bonanno S.L., Martin K.C., Khakh B.S. (2018). An Optical Neuron-Astrocyte Proximity Assay at Synaptic Distance Scales. Neuron.

[86] Martín R., Bajo-Grañeras R., Moratalla R., Perea G., Araque A. (2015). Circuit-specific signaling in astrocyte-neuron networks in basal ganglia pathways. Science.

[87] Cavaccini A., Durkee C., Kofuji P., Tonini R., Araque A. (2020). Astrocyte Signaling Gates Long-Term Depression at Corticostriatal Synapses of the Direct Pathway. J. Neurosci..

[88] Valtcheva S., Venance L. (2016). Astrocytes gate Hebbian synaptic plasticity in the striatum. Nat. Commun..

[89] Nagai J., Rajbhandari A.K., Gangwani M.R., Hachisuka A., Coppola G., Masmanidis S.C., Fanselow M.S., Khakh B.S. (2019). Hyperactivity with Disrupted Attention by Activation of an Astrocyte Synaptogenic Cue. Cell.

[90] De Biase L.M., Schuebel K.E., Fusfeld Z.H., Jair K., Hawes I.A., Cimbro R., Zhang H.-Y., Liu Q.-R., Shen H., Xi Z.-X. (2017). Local Cues Establish and Maintain Region-Specific Phenotypes of Basal Ganglia Microglia. Neuron.

[91] Kopec A.M., Smith C.J., Ayre N.R., Sweat S.C., Bilbo S.D. (2018). Microglial dopamine receptor elimination defines sex-specific nucleus accumbens development and social behavior in adolescent rats. Nat. Commun..

[92] Kashima D.T., Grueter B.A. (2017). Toll-like receptor 4 deficiency alters nucleus accumbens synaptic physiology and drug reward behavior. Proc. Natl. Acad. Sci. USA.

[93] Rizzo F.R., Musella A., De Vito F., Fresegna D., Bullitta S., Vanni V., Guadalupi L., Stampanoni Bassi M., Buttari F., Mandolesi G. (2018). Tumor Necrosis Factor and Interleukin-1β Modulate Synaptic Plasticity during Neuroinflammation. Neural Plast..

[94] Lewitus G.M., Pribiag H., Duseja R., St-Hilaire M., Stellwagen D. (2014). An adaptive role of TNFα in the regulation of striatal synapses. J. Neurosci..

[95] Mogi M., Togari A., Tanaka K., Ogawa N., Ichinose H., Nagatsu T. (1999). Increase in level of tumor necrosis factor (TNF)-alpha in 6-hydroxydopamine-lesioned striatum in rats without influence of systemic L-DOPA on the TNF-alpha induction. Neurosci. Lett..

[96] Miller B.J., Buckley P., Seabolt W., Mellor A., Kirkpatrick B. (2011). Meta-analysis of cytokine alterations in schizophrenia: Clinical status and antipsychotic effects. Biol. Psychiatry.

[97] Shaw B.C., Anders V.R., Tinkey R.A., Habean M.L., Brock O.D., Frostino B.J., Williams J.L. (2024). Immunity impacts cognitive deficits across neurological disorders. J. Neurochem..

[98] Michels S., Mali A., Jäntti H., Rezaie M., Malm T. (2025). Microglial involvement in autism spectrum disorder: Insights from human data and iPSC models. Brain Behav. Immun..

[99] Mohandas E., Rajmohan V., Raghunath B. (2009). Neurobiology of Alzheimer’s disease. Indian J. Psychiatry.

[100] McKhann G.M., Knopman D.S., Chertkow H., Hyman B.T., Jack C.R., Kawas C.H., Klunk W.E., Koroshetz W.J., Manly J.J., Mayeux R. (2011). The diagnosis of dementia due to Alzheimer’s disease: Recommendations from the National Institute on Aging-Alzheimer’s Association workgroups on diagnostic guidelines for Alzheimer’s disease. Alzheimer’s Dement..

[101] Fakorede S., Lateef O.M., Garuba W.A., Akosile P.O., Okon D.A., Aborode A.T. (2025). Dual impact of neuroinflammation on cognitive and motor impairments in Alzheimer’s disease. J. Alzheimer’s Dis. Rep..

[102] Sobue A., Komine O., Yamanaka K. (2023). Neuroinflammation in Alzheimer’s disease: Microglial signature and their relevance to disease. Inflamm. Regen..

[103] Ishii T., Haga S. (1975). Identification of components of immunoglobulins in senile plaques by means of fluorescent antibody technique. Acta Neuropathol..

[104] Rogers J., Luber-Narod J., Styren S.D., Civin W.H. (1988). Expression of immune system-associated antigens by cells of the human central nervous system: Relationship to the pathology of Alzheimer’s disease. Neurobiol. Aging.

[105] Duffy P.E., Rapport M., Graf L. (1980). Glial fibrillary acidic protein and Alzheimer-type senile dementia. Neurology.

[106] McGeer P.L., Itagaki S., Boyes B.E., McGeer E.G. (1988). Reactive microglia are positive for HLA-DR in the substantia nigra of Parkinson’s and Alzheimer’s disease brains. Neurology.

[107] Rich J.B., Rasmusson D.X., Folstein M.F., Carson K.A., Kawas C., Brandt J. (1995). Nonsteroidal anti-inflammatory drugs in Alzheimer’s disease. Neurology.

[108] Lagarde J., Sarazin M., Bottlaender M. (2018). In vivo PET imaging of neuroinflammation in Alzheimer’s disease. J. Neural Transm..

[109] Holmes C., Cunningham C., Zotova E., Woolford J., Dean C., Kerr S., Culliford D., Perry V.H. (2009). Systemic inflammation and disease progression in Alzheimer disease. Neurology.

[110] Park S.-H., Lee E.-H., Kim H.-J., Jo S., Lee S., Seo S.W., Park H.-H., Koh S.-H., Lee J.-H. (2021). The relationship of soluble TREM2 to other biomarkers of sporadic Alzheimer’s disease. Sci. Rep..

[111] Pelkmans W., Shekari M., Brugulat-Serrat A., Sánchez-Benavides G., Minguillón C., Fauria K., Molinuevo J.L., Grau-Rivera O., González Escalante A., Kollmorgen G. (2024). Astrocyte biomarkers GFAP and YKL-40 mediate early Alzheimer’s disease progression. Alzheimer’s Dement..

[112] Fortea J., Pegueroles J., Alcolea D., Belbin O., Dols-Icardo O., Vaqué-Alcázar L., Videla L., Gispert J.D., Suárez-Calvet M., Johnson S.C. (2024). APOE4 homozygozity represents a distinct genetic form of Alzheimer’s disease. Nat. Med..

[113] Clayton K.A., Van Enoo A.A., Ikezu T. (2017). Alzheimer’s disease: The role of microglia in brain homeostasis and proteopathy. Front. Neurosci..

[114] Zheng C., Zhou X.-W., Wang J.-Z. (2016). The dual roles of cytokines in Alzheimer’s disease: Update on interleukins, TNF-α, TGF-β and IFN-γ. Transl. Neurodegener..

[115] McQuade A., Blurton-Jones M. (2019). Microglia in alzheimer’s disease: Exploring how genetics and phenotype influence risk. J. Mol. Biol..

[116] Sayas C.L., Ávila J. (2021). GSK-3 and Tau: A Key Duet in Alzheimer’s Disease. Cells.

[117] Chang N.P., DaPrano E.M., Lindman M., Estevez I., Chou T.W., Evans W.R., Nissenbaum M., McCourt M., Alzate D., Atkins C. (2024). Neuronal DAMPs exacerbate neurodegeneration via astrocytic RIPK3 signaling. JCI Insight.

[118] Condello C., Yuan P., Schain A., Grutzendler J. (2015). Microglia constitute a barrier that prevents neurotoxic protofibrillar Aβ42 hotspots around plaques. Nat. Commun..

[119] Verkhratsky A., Marutle A., Rodríguez-Arellano J.J., Nordberg A. (2015). Glial asthenia and functional paralysis: A new perspective on neurodegeneration and alzheimer’s disease. Neuroscientist.

[120] Verkhratsky A., Olabarria M., Noristani H.N., Yeh C.-Y., Rodriguez J.J. (2010). Astrocytes in Alzheimer’s disease. Neurotherapeutics.

[121] Verkhratsky A., Parpura V. (2016). Astrogliopathology in neurological, neurodevelopmental and psychiatric disorders. Neurobiol. Dis..

[122] La Barbera L., Krashia P., Loffredo G., Cauzzi E., De Paolis M.L., Montanari M., Saba L., Spoleti E., Ficchì S., Zaccone C. (2025). Midbrain degeneration triggers astrocyte reactivity and tau pathology in experimental Alzheimer’s Disease. Mol. Neurodegener..

[123] Frost G.R., Li Y.-M. (2017). The role of astrocytes in amyloid production and Alzheimer’s disease. Open Biol..

[124] Amelimojarad M., Amelimojarad M., Cui X. (2024). The emerging role of brain neuroinflammatory responses in Alzheimer’s disease. Front. Aging Neurosci..

[125] Wang W.-Y., Tan M.-S., Yu J.-T., Tan L. (2015). Role of pro-inflammatory cytokines released from microglia in Alzheimer’s disease. Ann. Transl. Med..

[126] Imbriani P., Martella G., Bonsi P., Pisani A. (2022). Oxidative stress and synaptic dysfunction in rodent models of Parkinson’s disease. Neurobiol. Dis..

[127] Ganguly U., Kaur U., Chakrabarti S.S., Sharma P., Agrawal B.K., Saso L., Chakrabarti S. (2021). Oxidative stress, neuroinflammation, and NADPH oxidase: Implications in the pathogenesis and treatment of alzheimer’s disease. Oxidative Med. Cell. Longev..

[128] Snyder E.M., Nong Y., Almeida C.G., Paul S., Moran T., Choi E.Y., Nairn A.C., Salter M.W., Lombroso P.J., Gouras G.K. (2005). Regulation of NMDA receptor trafficking by amyloid-beta. Nat. Neurosci..

[129] Huang W.-J., Zhang X., Chen W.-W. (2016). Role of oxidative stress in Alzheimer’s disease. Biomed. Rep..

[130] Meftah S., Gan J. (2023). Alzheimer’s disease as a synaptopathy: Evidence for dysfunction of synapses during disease progression. Front. Synaptic Neurosci..

[131] Shankar G.M., Walsh D.M. (2009). Alzheimer’s disease: Synaptic dysfunction and Abeta. Mol. Neurodegener..

[132] Li S., Jin M., Koeglsperger T., Shepardson N.E., Shankar G.M., Selkoe D.J. (2011). Soluble Aβ oligomers inhibit long-term potentiation through a mechanism involving excessive activation of extrasynaptic NR2B-containing NMDA receptors. J. Neurosci..

[133] Forner S., Baglietto-Vargas D., Martini A.C., Trujillo-Estrada L., LaFerla F.M. (2017). Synaptic impairment in alzheimer’s disease: A dysregulated symphony. Trends Neurosci..

[134] Benarroch E.E. (2018). Glutamatergic synaptic plasticity and dysfunction in Alzheimer disease: Emerging mechanisms. Neurology.

[135] Zhang H., Jiang X., Ma L., Wei W., Li Z., Chang S., Wen J., Sun J., Li H. (2022). Role of Aβ in Alzheimer’s-related synaptic dysfunction. Front. Cell Dev. Biol..

[136] Zhang Y., Chen H., Li R., Sterling K., Song W. (2023). Amyloid β-based therapy for Alzheimer’s disease: Challenges, successes and future. Signal Transduct. Target. Ther..

[137] Prieto G.A., Tong L., Smith E.D., Cotman C.W. (2019). TNFα and IL-1β but not IL-18 Suppresses Hippocampal Long-Term Potentiation Directly at the Synapse. Neurochem. Res..

[138] Li S., Selkoe D.J. (2020). A mechanistic hypothesis for the impairment of synaptic plasticity by soluble Aβ oligomers from Alzheimer’s brain. J. Neurochem..

[139] Gomez-Arboledas A., Acharya M.M., Tenner A.J. (2021). The role of complement in synaptic pruning and neurodegeneration. Immunotargets Ther..

[140] Hong S., Beja-Glasser V.F., Nfonoyim B.M., Frouin A., Li S., Ramakrishnan S., Merry K.M., Shi Q., Rosenthal A., Barres B.A. (2016). Complement and microglia mediate early synapse loss in Alzheimer mouse models. Science.

[141] Gao C., Jiang J., Tan Y., Chen S. (2023). Microglia in neurodegenerative diseases: Mechanism and potential therapeutic targets. Signal Transduct. Target. Ther..

[142] Mecca A.P., Ashton N.J., Chen M.-K., O’Dell R.S., Toyonaga T., Zhao W., Young J.J., Salardini E., Bates K.A., Ra J. (2025). Cerebrospinal fluid and brain positron emission tomography measures of synaptic vesicle glycoprotein 2A: Biomarkers of synaptic density in Alzheimer’s disease. Alzheimer’s Dement..

[143] Bavarsad M.S., Grinberg L.T. (2024). SV2A PET imaging in human neurodegenerative diseases. Front. Aging Neurosci..

[144] Fang X.T., Raval N.R., O’Dell R.S., Naganawa M., Mecca A.P., Chen M.-K., van Dyck C.H., Carson R.E. (2024). Synaptic density patterns in early Alzheimer’s disease assessed by independent component analysis. Brain Commun..

[145] Kester M.I., Teunissen C.E., Crimmins D.L., Herries E.M., Ladenson J.H., Scheltens P., van der Flier W.M., Morris J.C., Holtzman D.M., Fagan A.M. (2015). Neurogranin as a cerebrospinal fluid biomarker for synaptic loss in symptomatic Alzheimer disease. JAMA Neurol..

[146] Agnello L., Lo Sasso B., Vidali M., Scazzone C., Piccoli T., Gambino C.M., Bivona G., Giglio R.V., Ciaccio A.M., La Bella V. (2021). Neurogranin as a reliable biomarker for synaptic dysfunction in Alzheimer’s disease. Diagnostics.

[147] Agnello L., Gambino C.M., Ciaccio A.M., Cacciabaudo F., Massa D., Masucci A., Tamburello M., Vassallo R., Midiri M., Scazzone C. (2025). From Amyloid to Synaptic Dysfunction: Biomarker-Driven Insights into Alzheimer’s Disease. Curr. Issues Mol. Biol..

[148] Nilsson J., Gobom J., Sjödin S., Brinkmalm G., Ashton N.J., Svensson J., Johansson P., Portelius E., Zetterberg H., Blennow K. (2021). Cerebrospinal fluid biomarker panel for synaptic dysfunction in Alzheimer’s disease. Alzheimer’s Dement..

[149] Dorsey E.R., Bloem B.R. (2018). The Parkinson Pandemic-A Call to Action. JAMA Neurol..

[150] Poewe W., Seppi K., Tanner C.M., Halliday G.M., Brundin P., Volkmann J., Schrag A.-E., Lang A.E. (2017). Parkinson disease. Nat. Rev. Dis. Primers.

[151] Kalia L.V., Lang A.E. (2015). Parkinson’s disease. Lancet.

[152] Ma J., Gao J., Wang J., Xie A. (2019). Prion-Like Mechanisms in Parkinson’s Disease. Front. Neurosci..

[153] Nguyen M., Wong Y.C., Ysselstein D., Severino A., Krainc D. (2019). Synaptic, mitochondrial, and lysosomal dysfunction in Parkinson’s disease. Trends Neurosci..

[154] Lawson L.J., Perry V.H., Dri P., Gordon S. (1990). Heterogeneity in the distribution and morphology of microglia in the normal adult mouse brain. Neuroscience.

[155] Kuter K., Olech Ł., Głowacka U. (2018). Prolonged Dysfunction of Astrocytes and Activation of Microglia Accelerate Degeneration of Dopaminergic Neurons in the Rat Substantia Nigra and Block Compensation of Early Motor Dysfunction Induced by 6-OHDA. Mol. Neurobiol..

[156] Zecca L., Casella L., Albertini A., Bellei C., Zucca F.A., Engelen M., Zadlo A., Szewczyk G., Zareba M., Sarna T. (2008). Neuromelanin can protect against iron-mediated oxidative damage in system modeling iron overload of brain aging and Parkinson’s disease. J. Neurochem..

[157] Hirsch E.C., Vyas S., Hunot S. (2012). Neuroinflammation in Parkinson’s disease. Park. Relat. Disord..

[158] Ben-Shachar D., Riederer P., Youdim M.B. (1991). Iron-melanin interaction and lipid peroxidation: Implications for Parkinson’s disease. J. Neurochem..

[159] Thomas Broome S., Louangaphay K., Keay K.A., Leggio G.M., Musumeci G., Castorina A. (2020). Dopamine: An immune transmitter. Neural Regen. Res..

[160] Arreola R., Alvarez-Herrera S., Pérez-Sánchez G., Becerril-Villanueva E., Cruz-Fuentes C., Flores-Gutierrez E.O., Garcés-Alvarez M.E., de la Cruz-Aguilera D.L., Medina-Rivero E., Hurtado-Alvarado G. (2016). Immunomodulatory effects mediated by dopamine. J. Immunol. Res..

[161] Joers V., Tansey M.G., Mulas G., Carta A.R. (2017). Microglial phenotypes in Parkinson’s disease and animal models of the disease. Prog. Neurobiol..

[162] Zimmermann M., Brockmann K. (2022). Blood and cerebrospinal fluid biomarkers of inflammation in parkinson’s disease. J. Parkinson’s Dis..

[163] Imamura K., Hishikawa N., Sawada M., Nagatsu T., Yoshida M., Hashizume Y. (2003). Distribution of major histocompatibility complex class II-positive microglia and cytokine profile of Parkinson’s disease brains. Acta Neuropathol..

[164] Weiss F., Labrador-Garrido A., Dzamko N., Halliday G. (2022). Immune responses in the Parkinson’s disease brain. Neurobiol. Dis..

[165] Pajares M., I Rojo A., Manda G., Boscá L., Cuadrado A. (2020). Inflammation in parkinson’s disease: Mechanisms and therapeutic implications. Cells.

[166] Belloli S., Morari M., Murtaj V., Valtorta S., Moresco R.M., Gilardi M.C. (2020). Translation imaging in parkinson’s disease: Focus on neuroinflammation. Front. Aging Neurosci..

[167] Mirza B., Hadberg H., Thomsen P., Moos T. (2000). The absence of reactive astrocytosis is indicative of a unique inflammatory process in Parkinson’s disease. Neuroscience.

[168] Tong J., Ang L.-C., Williams B., Furukawa Y., Fitzmaurice P., Guttman M., Boileau I., Hornykiewicz O., Kish S.J. (2015). Low levels of astroglial markers in Parkinson’s disease: Relationship to α-synuclein accumulation. Neurobiol. Dis..

[169] Morales I., Sanchez A., Rodriguez-Sabate C., Rodriguez M. (2016). The astrocytic response to the dopaminergic denervation of the striatum. J. Neurochem..

[170] Zhu X., Grace A.A. (2021). Prepubertal environmental enrichment prevents dopamine dysregulation and hippocampal hyperactivity in MAM schizophrenia model rats. Biol. Psychiatry.

[171] Simon D.K., Tanner C.M., Brundin P. (2020). Parkinson disease epidemiology, pathology, genetics, and pathophysiology. Clin. Geriatr. Med..

[172] Boyd R.J., Avramopoulos D., Jantzie L.L., McCallion A.S. (2022). Neuroinflammation represents a common theme amongst genetic and environmental risk factors for Alzheimer and Parkinson diseases. J. Neuroinflamm..

[173] Blandini F., Armentero M.-T. (2012). Animal models of Parkinson’s disease. FEBS J..

[174] Bezard E., Yue Z., Kirik D., Spillantini M.G. (2013). Animal models of Parkinson’s disease: Limits and relevance to neuroprotection studies. Mov. Disord..

[175] Langston J.W., Ballard P., Tetrud J.W., Irwin I. (1983). Chronic Parkinsonism in humans due to a product of meperidine-analog synthesis. Science.

[176] Kim-Han J.S., Antenor-Dorsey J.A., O’Malley K.L. (2011). The parkinsonian mimetic, MPP+, specifically impairs mitochondrial transport in dopamine axons. J. Neurosci..

[177] Glass C.K., Saijo K., Winner B., Marchetto M.C., Gage F.H. (2010). Mechanisms underlying inflammation in neurodegeneration. Cell.

[178] Bu X.-L., Wang X., Xiang Y., Shen L.-L., Wang Q.-H., Liu Y.-H., Jiao S.-S., Wang Y.-R., Cao H.-Y., Yi X. (2015). The association between infectious burden and Parkinson’s disease: A case-control study. Park. Relat. Disord..

[179] Qin L., Wu X., Block M.L., Liu Y., Breese G.R., Hong J.-S., Knapp D.J., Crews F.T. (2007). Systemic LPS causes chronic neuroinflammation and progressive neurodegeneration. Glia.

[180] Dzamko N., Geczy C.L., Halliday G.M. (2015). Inflammation is genetically implicated in Parkinson’s disease. Neuroscience.

[181] Chao Y., Wong S.C., Tan E.K. (2014). Evidence of inflammatory system involvement in Parkinson’s disease. Biomed. Res. Int..

[182] Gagliano S.A., Pouget J.G., Hardy J., Knight J., Barnes M.R., Ryten M., Weale M.E. (2016). Genomics implicates adaptive and innate immunity in Alzheimer’s and Parkinson’s diseases. Ann. Clin. Transl. Neurol..

[183] Tolosa E., Vila M., Klein C., Rascol O. (2020). LRRK2 in Parkinson disease: Challenges of clinical trials. Nat. Rev. Neurol..

[184] Mancini A., Mazzocchetti P., Sciaccaluga M., Megaro A., Bellingacci L., Beccano-Kelly D.A., Di Filippo M., Tozzi A., Calabresi P. (2020). From synaptic dysfunction to neuroprotective strategies in genetic parkinson’s disease: Lessons from LRRK2. Front. Cell. Neurosci..

[185] Wallings R., Manzoni C., Bandopadhyay R. (2015). Cellular processes associated with LRRK2 function and dysfunction. FEBS J..

[186] Kim B., Yang M.-S., Choi D., Kim J.-H., Kim H.-S., Seol W., Choi S., Jou I., Kim E.-Y., Joe E.-H. (2012). Impaired inflammatory responses in murine Lrrk2-knockdown brain microglia. PLoS ONE.

[187] Ma B., Xu L., Pan X., Sun L., Ding J., Xie C., Koliatsos V.E., Cai H. (2016). LRRK2 modulates microglial activity through regulation of chemokine (C-X3-C) receptor 1 -mediated signalling pathways. Hum. Mol. Genet..

[188] Puccini J.M., Marker D.F., Fitzgerald T., Barbieri J., Kim C.S., Miller-Rhodes P., Lu S.-M., Dewhurst S., Gelbard H.A. (2015). Leucine-rich repeat kinase 2 modulates neuroinflammation and neurotoxicity in models of human immunodeficiency virus 1-associated neurocognitive disorders. J. Neurosci..

[189] Farrer M., Kachergus J., Forno L., Lincoln S., Wang D.-S., Hulihan M., Maraganore D., Gwinn-Hardy K., Wszolek Z., Dickson D. (2004). Comparison of kindreds with parkinsonism and alpha-synuclein genomic multiplications. Ann. Neurol..

[190] Cardinale A., Calabrese V., de Iure A., Picconi B. (2021). Alpha-Synuclein as a Prominent Actor in the Inflammatory Synaptopathy of Parkinson’s Disease. Int. J. Mol. Sci..

[191] Longhena F., Faustini G., Missale C., Pizzi M., Spano P., Bellucci A. (2017). The Contribution of α-Synuclein Spreading to Parkinson’s Disease Synaptopathy. Neural Plast..

[192] Diógenes M.J., Dias R.B., Rombo D.M., Vicente Miranda H., Maiolino F., Guerreiro P., Näsström T., Franquelim H.G., Oliveira L.M.A., Castanho M.A.R.B. (2012). Extracellular alpha-synuclein oligomers modulate synaptic transmission and impair LTP via NMDA-receptor activation. J. Neurosci..

[193] Tozzi A., de Iure A., Bagetta V., Tantucci M., Durante V., Quiroga-Varela A., Costa C., Di Filippo M., Ghiglieri V., Latagliata E.C. (2016). Alpha-Synuclein Produces Early Behavioral Alterations via Striatal Cholinergic Synaptic Dysfunction by Interacting with GluN2D N-Methyl-D-Aspartate Receptor Subunit. Biol. Psychiatry.

[194] Durante V., de Iure A., Loffredo V., Vaikath N., De Risi M., Paciotti S., Quiroga-Varela A., Chiasserini D., Mellone M., Mazzocchetti P. (2019). Alpha-synuclein targets GluN2A NMDA receptor subunit causing striatal synaptic dysfunction and visuospatial memory alteration. Brain.

[195] Hoffmann A., Ettle B., Bruno A., Kulinich A., Hoffmann A.-C., von Wittgenstein J., Winkler J., Xiang W., Schlachetzki J.C.M. (2016). Alpha-synuclein activates BV2 microglia dependent on its aggregation state. Biochem. Biophys. Res. Commun..

[196] Braak H., Del Tredici K., Rüb U., de Vos R.A.I., Jansen Steur E.N.H., Braak E. (2003). Staging of brain pathology related to sporadic Parkinson’s disease. Neurobiol. Aging.

[197] Jiang T., Hoekstra J., Heng X., Kang W., Ding J., Liu J., Chen S., Zhang J. (2015). P2X7 receptor is critical in α-synuclein--mediated microglial NADPH oxidase activation. Neurobiol. Aging.

[198] Sidransky E., Nalls M.A., Aasly J.O., Aharon-Peretz J., Annesi G., Barbosa E.R., Bar-Shira A., Berg D., Bras J., Brice A. (2009). Multicenter analysis of glucocerebrosidase mutations in Parkinson’s disease. N. Engl. J. Med..

[199] Mullin S., Stokholm M.G., Hughes D., Mehta A., Parbo P., Hinz R., Pavese N., Brooks D.J., Schapira A.H.V. (2021). Brain Microglial Activation Increased in Glucocerebrosidase (GBA) Mutation Carriers without Parkinson’s disease. Mov. Disord..

[200] Smith L., Schapira A.H.V. (2022). GBA variants and parkinson disease: Mechanisms and treatments. Cells.

[201] Hughes L.P., Pereira M.M.M., Hammond D.A., Kwok J.B., Halliday G.M., Lewis S.J.G., Dzamko N. (2021). Glucocerebrosidase Activity is Reduced in Cryopreserved Parkinson’s Disease Patient Monocytes and Inversely Correlates with Motor Severity. J. Parkinson’s Dis..

[202] Chahine L.M., Qiang J., Ashbridge E., Minger J., Yearout D., Horn S., Colcher A., Hurtig H.I., Lee V.M.-Y., Van Deerlin V.M. (2013). Clinical and biochemical differences in patients having Parkinson disease with vs without GBA mutations. JAMA Neurol..

[203] Kam T.-I., Hinkle J.T., Dawson T.M., Dawson V.L. (2020). Microglia and astrocyte dysfunction in parkinson’s disease. Neurobiol. Dis..

[204] Valente E.M., Abou-Sleiman P.M., Caputo V., Muqit M.M.K., Harvey K., Gispert S., Ali Z., Del Turco D., Bentivoglio A.R., Healy D.G. (2004). Hereditary early-onset Parkinson’s disease caused by mutations in PINK1. Science.

[205] Kitada T., Asakawa S., Hattori N., Matsumine H., Yamamura Y., Minoshima S., Yokochi M., Mizuno Y., Shimizu N. (1998). Mutations in the parkin gene cause autosomal recessive juvenile parkinsonism. Nature.

[206] Pickrell A.M., Youle R.J. (2015). The roles of PINK1, parkin, and mitochondrial fidelity in Parkinson’s disease. Neuron.

[207] Borsche M., König I.R., Delcambre S., Petrucci S., Balck A., Brüggemann N., Zimprich A., Wasner K., Pereira S.L., Avenali M. (2020). Mitochondrial damage-associated inflammation highlights biomarkers in PRKN/PINK1 parkinsonism. Brain.

[208] Gustin A., Kirchmeyer M., Koncina E., Felten P., Losciuto S., Heurtaux T., Tardivel A., Heuschling P., Dostert C. (2015). NLRP3 inflammasome is expressed and functional in mouse brain microglia but not in astrocytes. PLoS ONE.

[209] Yan Y.-Q., Zheng R., Liu Y., Ruan Y., Lin Z.-H., Xue N.-J., Chen Y., Zhang B.-R., Pu J.-L. (2023). Parkin regulates microglial NLRP3 and represses neurodegeneration in Parkinson’s disease. Aging Cell.

[210] Soman S.K., Dagda R.K. (2021). Role of cleaved PINK1 in neuronal development, synaptogenesis, and plasticity: Implications for parkinson’s disease. Front. Neurosci..

[211] Imbriani P., Schirinzi T., Meringolo M., Mercuri N.B., Pisani A. (2018). Centrality of early synaptopathy in parkinson’s disease. Front. Neurol..

[212] Petrelli F., Pucci L., Bezzi P. (2016). Astrocytes and Microglia and Their Potential Link with Autism Spectrum Disorders. Front. Cell. Neurosci..

[213] Li Q., Barres B.A. (2018). Microglia and macrophages in brain homeostasis and disease. Nat. Rev. Immunol..

[214] Matta S.M., Hill-Yardin E.L., Crack P.J. (2019). The influence of neuroinflammation in Autism Spectrum Disorder. Brain Behav. Immun..

[215] Maenner M.J., Shaw K.A., Bakian A.V., Bilder D.A., Durkin M.S., Esler A., Furnier S.M., Hallas L., Hall-Lande J., Hudson A. (2021). Prevalence and Characteristics of Autism Spectrum Disorder Among Children Aged 8 Years—Autism and Developmental Disabilities Monitoring Network, 11 Sites, United States, 2018. MMWR Surveill. Summ..

[216] Gaugler T., Klei L., Sanders S.J., Bodea C.A., Goldberg A.P., Lee A.B., Mahajan M., Manaa D., Pawitan Y., Reichert J. (2014). Most genetic risk for autism resides with common variation. Nat. Genet..

[217] Hughes H.K., Moreno R.J., Ashwood P. (2024). Innate immune dysfunction and neuroinflammation in autism spectrum disorder (ASD). Focus.

[218] Enstrom A.M., Onore C.E., Van de Water J.A., Ashwood P. (2010). Differential monocyte responses to TLR ligands in children with autism spectrum disorders. Brain Behav. Immun..

[219] Tural Hesapcioglu S., Kasak M., Cıtak Kurt A.N., Ceylan M.F. (2017). High monocyte level and low lymphocyte to monocyte ratio in autism spectrum disorders. Int. J. Dev. Disabil..

[220] Ashwood P., Krakowiak P., Hertz-Picciotto I., Hansen R., Pessah I., Van de Water J. (2011). Elevated plasma cytokines in autism spectrum disorders provide evidence of immune dysfunction and are associated with impaired behavioral outcome. Brain Behav. Immun..

[221] Vargas D.L., Nascimbene C., Krishnan C., Zimmerman A.W., Pardo C.A. (2005). Neuroglial activation and neuroinflammation in the brain of patients with autism. Ann. Neurol..

[222] Morgan J.T., Chana G., Abramson I., Semendeferi K., Courchesne E., Everall I.P. (2012). Abnormal microglial-neuronal spatial organization in the dorsolateral prefrontal cortex in autism. Brain Res..

[223] Liao X., Yang J., Wang H., Li Y. (2020). Microglia mediated neuroinflammation in autism spectrum disorder. J. Psychiatr. Res..

[224] Zhan Y., Paolicelli R.C., Sforazzini F., Weinhard L., Bolasco G., Pagani F., Vyssotski A.L., Bifone A., Gozzi A., Ragozzino D. (2014). Deficient neuron-microglia signaling results in impaired functional brain connectivity and social behavior. Nat. Neurosci..

[225] Paolicelli R.C., Bolasco G., Pagani F., Maggi L., Scianni M., Panzanelli P., Giustetto M., Ferreira T.A., Guiducci E., Dumas L. (2011). Synaptic pruning by microglia is necessary for normal brain development. Science.

[226] Thion M.S., Mosser C.-A., Férézou I., Grisel P., Baptista S., Low D., Ginhoux F., Garel S., Audinat E. (2019). Biphasic impact of prenatal inflammation and macrophage depletion on the wiring of neocortical inhibitory circuits. Cell Rep..

[227] Traetta M.E., Uccelli N.A., Zárate S.C., Gómez Cuautle D., Ramos A.J., Reinés A. (2021). Long-Lasting Changes in Glial Cells Isolated From Rats Subjected to the Valproic Acid Model of Autism Spectrum Disorder. Front. Pharmacol..

[228] Tian Y., Xiao X., Liu W., Cheng S., Qian N., Wang L., Liu Y., Ai R., Zhu X. (2024). TREM2 improves microglia function and synaptic development in autism spectrum disorders by regulating P38 MAPK signaling pathway. Mol. Brain.

[229] Gussago C., Casati M., Ferri E., Arosio B. (2019). The Triggering Receptor Expressed on Myeloid Cells-2 (TREM-2) as Expression of the Relationship between Microglia and Alzheimer’s Disease: A Novel Marker for a Promising Pathway to Explore. J. Frailty Aging.

[230] Luo L., Chen J., Wu Q., Yuan B., Hu C., Yang T., Wei H., Li T. (2023). Prenatally VPA exposure is likely to cause autistic-like behavior in the rats offspring via TREM2 down-regulation to affect the microglial activation and synapse alterations. Environ. Toxicol. Pharmacol..

[231] Cieślik M., Gąssowska-Dobrowolska M., Jęśko H., Czapski G.A., Wilkaniec A., Zawadzka A., Dominiak A., Polowy R., Filipkowski R.K., Boguszewski P.M. (2020). Maternal immune activation induces neuroinflammation and cortical synaptic deficits in the adolescent rat offspring. Int. J. Mol. Sci..

[232] Jiang J., Zhang L., Wu D., Zhao D., Ying S., Ding S. (2025). Lipopolysaccharide induces neuroinflammation in a valproic acid male model of autism. Brain Res. Bull..

[233] Chen H.-R., Chen C.-W., Mandhani N., Short-Miller J.C., Smucker M.R., Sun Y.-Y., Kuan C.-Y. (2020). Monocytic Infiltrates Contribute to Autistic-like Behaviors in a Two-Hit Model of Neurodevelopmental Defects. J. Neurosci..

[234] Uchigashima M., Cheung A., Futai K. (2021). Neuroligin-3: A Circuit-Specific Synapse Organizer That Shapes Normal Function and Autism Spectrum Disorder-Associated Dysfunction. Front. Mol. Neurosci..

[235] Rothwell P.E., Fuccillo M.V., Maxeiner S., Hayton S.J., Gokce O., Lim B.K., Fowler S.C., Malenka R.C., Südhof T.C. (2014). Autism-associated neuroligin-3 mutations commonly impair striatal circuits to boost repetitive behaviors. Cell.

[236] Meringolo M., Montanari M., D’Antoni S., Martella G., El Atiallah I., Ponterio G., Tassone A., Reverte I., Caprioli D., Strimpakos G. (2025). Impairment of Group I Metabotropic Glutamate Receptors in the Dorsal Striatum of the *R*451C-Neuroligin 3 Mouse Model of Autism Spectrum Disorder. J. Neurochem..

[237] Matta S.M., Moore Z., Walker F.R., Hill-Yardin E.L., Crack P.J. (2020). An altered glial phenotype in the NL3R451C mouse model of autism. Sci. Rep..

[238] Martella G., Meringolo M., Trobiani L., De Jaco A., Pisani A., Bonsi P. (2018). The neurobiological bases of autism spectrum disorders: The R451C-neuroligin 3 mutation hampers the expression of long-term synaptic depression in the dorsal striatum. Eur. J. Neurosci..

[239] Guy J., Hendrich B., Holmes M., Martin J.E., Bird A. (2001). A mouse Mecp2-null mutation causes neurological symptoms that mimic Rett syndrome. Nat. Genet..

[240] Maezawa I., Jin L.-W. (2010). Rett syndrome microglia damage dendrites and synapses by the elevated release of glutamate. J. Neurosci..

[241] Maezawa I., Swanberg S., Harvey D., LaSalle J.M., Jin L.-W. (2009). Rett syndrome astrocytes are abnormal and spread MeCP2 deficiency through gap junctions. J. Neurosci..

[242] Ballas N., Lioy D.T., Grunseich C., Mandel G. (2009). Non-cell autonomous influence of MeCP2-deficient glia on neuronal dendritic morphology. Nat. Neurosci..

[243] Jacobs S., Nathwani M., Doering L.C. (2010). Fragile X astrocytes induce developmental delays in dendrite maturation and synaptic protein expression. BMC Neurosci..

[244] Borreca A., Santamaria G., Matteoli M. (2023). Epigenetic mechanism affects microglia status and synaptic pruning mechanism in fragile x syndrome. IBRO Neurosci. Rep..

[245] Estes M.L., McAllister A.K. (2015). Immune mediators in the brain and peripheral tissues in autism spectrum disorder. Nat. Rev. Neurosci..

[246] Onore C., Careaga M., Ashwood P. (2012). The role of immune dysfunction in the pathophysiology of autism. Brain Behav. Immun..

[247] Masi A., Quintana D.S., Glozier N., Lloyd A.R., Hickie I.B., Guastella A.J. (2015). Cytokine aberrations in autism spectrum disorder: A systematic review and meta-analysis. Mol. Psychiatry.

[248] Di Benedetto S., Müller L., Wenger E., Düzel S., Pawelec G. (2017). Contribution of neuroinflammation and immunity to brain aging and the mitigating effects of physical and cognitive interventions. Neurosci. Biobehav. Rev..

[249] Liang L.-B., Wang S., Li K.-P., Wu C.-Q. (2025). Comparative efficacy of cognitive training modalities in cognitive impairment: A systematic review and network meta-analysis. J. Prev. Alzheimer’s Dis..

[250] Etnier J.L., Wessinger C.M., Herrera B.M., Kayser K.C. (2025). Chronic physical activity and the prevention of Alzheimer’s disease. Psychol. Sport Exerc..

[251] Langeskov-Christensen M., Franzén E., Grøndahl Hvid L., Dalgas U. (2024). Exercise as medicine in Parkinson’s disease. J. Neurol. Neurosurg. Psychiatry.

[252] Leung I.H.K., Walton C.C., Hallock H., Lewis S.J.G., Valenzuela M., Lampit A. (2015). Cognitive training in Parkinson disease: A systematic review and meta-analysis. Neurology.

[253] Jiang W., Wang X., Mao L. (2025). Effects of resistance exercise on cognitive function, neurotrophic factors, brain structure, and brain function in older adults: A narrative review. J. Alzheimer’s Dis..

[254] Marino G., Campanelli F., Natale G., De Carluccio M., Servillo F., Ferrari E., Gardoni F., Caristo M.E., Picconi B., Cardinale A. (2023). Intensive exercise ameliorates motor and cognitive symptoms in experimental Parkinson’s disease restoring striatal synaptic plasticity. Sci. Adv..

[255] Petzinger G.M., Fisher B.E., McEwen S., Beeler J.A., Walsh J.P., Jakowec M.W. (2013). Exercise-enhanced neuroplasticity targeting motor and cognitive circuitry in Parkinson’s disease. Lancet Neurol..

[256] Pinho R.A., Muller A.P., Marqueze L.F., Radak Z., Arida R.M. (2024). Physical exercise-mediated neuroprotective mechanisms in Parkinson’s disease, Alzheimer’s disease, and epilepsy. Braz. J. Med. Biol. Res..

[257] Kong J., Xie Y., Fan R., Wang Q., Luo Y., Dong P. (2025). Exercise orchestrates systemic metabolic and neuroimmune homeostasis via the brain-muscle-liver axis to slow down aging and neurodegeneration: A narrative review. Eur. J. Med. Res..

[258] Gobbi L.T.B., Pelicioni P.H.S., Lahr J., Lirani-Silva E., Teixeira-Arroyo C., Santos P.C.R.D. (2021). Effect of different types of exercises on psychological and cognitive features in people with Parkinson’s disease: A randomized controlled trial. Ann. Phys. Rehabil. Med..

[259] Kim Y., Oh W., You J.S.H. (2023). Immediate effects of multimodal cognitive therapy in mild cognitive impairment. NeuroRehabilitation.

[260] Stern Y. (2002). What is cognitive reserve? Theory and research application of the reserve concept. J. Int. Neuropsychol. Soc..

[261] Clare L., Wu Y.-T., Teale J.C., MacLeod C., Matthews F., Brayne C., Woods B., CFAS-Wales study team (2017). Potentially modifiable lifestyle factors, cognitive reserve, and cognitive function in later life: A cross-sectional study. PLoS Med..

[262] Pettigrew C., Soldan A. (2019). Defining cognitive reserve and implications for cognitive aging. Curr. Neurol. Neurosci. Rep..

[263] Serra L., Gelfo F. (2019). What good is the reserve? A translational perspective for the managing of cognitive decline. Neural Regen. Res..

[264] Kempermann G. (2019). Environmental enrichment, new neurons and the neurobiology of individuality. Nat. Rev. Neurosci..

[265] Nithianantharajah J., Hannan A.J. (2006). Enriched environments, experience-dependent plasticity and disorders of the nervous system. Nat. Rev. Neurosci..

[266] Gelfo F., Petrosini L. (2022). Environmental enrichment enhances cerebellar compensation and develops cerebellar reserve. Int. J. Environ. Res. Public Health.

[267] Gelfo F. (2019). Does experience enhance cognitive flexibility? an overview of the evidence provided by the environmental enrichment studies. Front. Behav. Neurosci..

[268] Landolfo E., Cutuli D., Decandia D., Balsamo F., Petrosini L., Gelfo F. (2023). Environmental Enrichment Protects against Neurotoxic Effects of Lipopolysaccharide: A Comprehensive Overview. Int. J. Mol. Sci..

[269] Vaquero-Rodríguez A., Ortuzar N., Lafuente J.V., Bengoetxea H. (2023). Enriched environment as a nonpharmacological neuroprotective strategy. Exp. Biol. Med..

[270] Jurgens H.A., Johnson R.W. (2012). Environmental enrichment attenuates hippocampal neuroinflammation and improves cognitive function during influenza infection. Brain Behav. Immun..

[271] Mandolesi L., Gelfo F., Serra L., Montuori S., Polverino A., Curcio G., Sorrentino G. (2017). Environmental Factors Promoting Neural Plasticity: Insights from Animal and Human Studies. Neural Plast..

[272] Popov A., Brazhe N., Morozova K., Yashin K., Bychkov M., Nosova O., Sutyagina O., Brazhe A., Parshina E., Li L. (2023). Mitochondrial malfunction and atrophy of astrocytes in the aged human cerebral cortex. Nat. Commun..

[273] Rodríguez J.J., Terzieva S., Olabarria M., Lanza R.G., Verkhratsky A. (2013). Enriched environment and physical activity reverse astrogliodegeneration in the hippocampus of AD transgenic mice. Cell Death Dis..

[274] Augusto-Oliveira M., Verkhratsky A. (2021). Mens sana in corpore sano: Lifestyle changes modify astrocytes to contain Alzheimer’s disease. Neural Regen. Res..

[275] Speisman R.B., Kumar A., Rani A., Pastoriza J.M., Severance J.E., Foster T.C., Ormerod B.K. (2013). Environmental enrichment restores neurogenesis and rapid acquisition in aged rats. Neurobiol. Aging.

[276] Herring A., Ambrée O., Tomm M., Habermann H., Sachser N., Paulus W., Keyvani K. (2009). Environmental enrichment enhances cellular plasticity in transgenic mice with Alzheimer-like pathology. Exp. Neurol..

[277] Hirase H., Shinohara Y. (2014). Transformation of cortical and hippocampal neural circuit by environmental enrichment. Neuroscience.

[278] de Oliveira T.C.G., Carvalho-Paulo D., de Lima C.M., de Oliveira R.B., Santos Filho C., Diniz D.G., Bento Torres Neto J., Picanço-Diniz C.W. (2020). Long-term environmental enrichment reduces microglia morphological diversity of the molecular layer of dentate gyrus. Eur. J. Neurosci..

[279] Sampedro-Piquero P., De Bartolo P., Petrosini L., Zancada-Menendez C., Arias J.L., Begega A. (2014). Astrocytic plasticity as a possible mediator of the cognitive improvements after environmental enrichment in aged rats. Neurobiol. Learn. Mem..

[280] He C., Tsipis C.P., LaManna J.C., Xu K. (2017). Environmental enrichment induces increased cerebral capillary density and improved cognitive function in mice. Adv. Exp. Med. Biol..

[281] Faherty C.J., Raviie Shepherd K., Herasimtschuk A., Smeyne R.J. (2005). Environmental enrichment in adulthood eliminates neuronal death in experimental Parkinsonism. Brain Res. Mol. Brain Res..

[282] Costa G.A., de Gusmão Taveiros Silva N.K., Marianno P., Chivers P., Bailey A., Camarini R. (2023). Environmental enrichment increased bdnf transcripts in the prefrontal cortex: Implications for an epigenetically controlled mechanism. Neuroscience.

[283] Cutuli D., Landolfo E., Petrosini L., Gelfo F. (2022). Environmental Enrichment Effects on the Brain-Derived Neurotrophic Factor Expression in Healthy Condition, Alzheimer’s Disease, and Other Neurodegenerative Disorders. J. Alzheimer’s Dis..

[284] Di Tella S., Isernia S., Cabinio M., Rossetto F., Borgnis F., Pagliari C., Cazzoli M., Navarro J., Silveri M.C., Baglio F. (2024). Cognitive Reserve proxies can modulate motor and non-motor basal ganglia circuits in early Parkinson’s Disease. Brain Imaging Behav..

[285] Guzzetti S., Mancini F., Caporali A., Manfredi L., Daini R. (2019). The association of cognitive reserve with motor and cognitive functions for different stages of Parkinson’s disease. Exp. Gerontol..

[286] Hindle J.V., Martin-Forbes P.A., Martyr A., Bastable A.J.M., Pye K.L., Mueller Gathercole V.C., Thomas E.M., Clare L. (2017). The effects of lifelong cognitive lifestyle on executive function in older people with Parkinson’s disease. Int. J. Geriatr. Psychiatry.

[287] Mantovani E., Bressan M.M., Tinazzi M., Tamburin S. (2024). Towards multimodal cognition-based treatment for cognitive impairment in Parkinson’s disease: Drugs, exercise, non-invasive brain stimulation and technologies. Curr. Opin. Neurol..

[288] Orgeta V., McDonald K.R., Poliakoff E., Hindle J.V., Clare L., Leroi I. (2020). Cognitive training interventions for dementia and mild cognitive impairment in Parkinson’s disease. Cochrane Database Syst. Rev..

[289] Walton C.C., Naismith S.L., Lampit A., Mowszowski L., Lewis S.J.G. (2017). Cognitive training in parkinson’s disease. Neurorehabil. Neural Repair.

[290] Ranieri A., Mennitti C., Falcone N., La Monica I., Di Iorio M.R., Tripodi L., Gentile A., Vitale M., Pero R., Pastore L. (2023). Positive effects of physical activity in autism spectrum disorder: How influences behavior, metabolic disorder and gut microbiota. Front. Psychiatry.

[291] Zhuang H., Liang Z., Ma G., Qureshi A., Ran X., Feng C., Liu X., Yan X., Shen L. (2024). Autism spectrum disorder: Pathogenesis, biomarker, and intervention therapy. MedComm.

[292] Toscano C.V.A., Barros L., Lima A.B., Nunes T., Carvalho H.M., Gaspar J.M. (2021). Neuroinflammation in autism spectrum disorders: Exercise as a “pharmacological” tool. Neurosci. Biobehav. Rev..

[293] Liu J.J., Wei Y.B., Strawbridge R., Bao Y., Chang S., Shi L., Que J., Gadad B.S., Trivedi M.H., Kelsoe J.R. (2020). Peripheral cytokine levels and response to antidepressant treatment in depression: A systematic review and meta-analysis. Mol. Psychiatry.

[294] Li Y., Lu J., Zhang J., Gui W., Xie W. (2024). Molecular insights into enriched environments and behavioral improvements in autism: A systematic review and meta-analysis. Front. Psychiatry.

[295] Farmer A.L., Lewis M.H. (2023). Reduction of restricted repetitive behavior by environmental enrichment: Potential neurobiological mechanisms. Neurosci. Biobehav. Rev..

[296] Woo C.C., Donnelly J.H., Steinberg-Epstein R., Leon M. (2015). Environmental enrichment as a therapy for autism: A clinical trial replication and extension. Behav. Neurosci..

[297] Marta K., Justyna C.-Ł., Marta S., Jerzy L., Justyna P.-B., Agnieszka B.-Z., Danuta O. (2022). Selected methods of therapeutic interactions with people with mild symptoms of autism spectrum disorder. Front. Psychiatry.

